# The mycotoxin viriditoxin induces leukemia- and lymphoma-specific apoptosis by targeting mitochondrial metabolism

**DOI:** 10.1038/s41419-022-05356-w

**Published:** 2022-11-08

**Authors:** Fabian Stuhldreier, Laura Schmitt, Thomas Lenz, Ilka Hinxlage, Marcel Zimmermann, Philipp Wollnitzke, Julian Schliehe-Diecks, Yang Liu, Paul Jäger, Stefanie Geyh, Nicole Teusch, Christoph Peter, Sanil Bhatia, Rainer Haas, Bodo Levkau, Andreas S. Reichert, Kai Stühler, Peter Proksch, Björn Stork, Sebastian Wesselborg

**Affiliations:** 1 Institute for Molecular Medicine I, Medical Faculty and University Hospital Düsseldorf, Heinrich Heine University, Universitätsstraße 1, 40225 Düsseldorf, Germany; 2 Molecular Proteomics Laboratory, Biological-Medical-Research Center (BMFZ), Medical Faculty and University Hospital Düsseldorf, Heinrich Heine University, Universitätsstraße 1, 40225 Düsseldorf, Germany; 3 Institute of Biochemistry and Molecular Biology I, Medical Faculty and University Hospital Düsseldorf, Heinrich Heine University, Universitätsstraße 1, 40225 Düsseldorf, Germany; 4 Institute for Molecular Medicine III, Medical Faculty and University Hospital Düsseldorf, Heinrich Heine University, Universitätsstraße 1, 40225 Düsseldorf, Germany; 5 Department of Pediatric Oncology, Hematology and Clinical Immunology, Medical Faculty and University Hospital Düsseldorf, Moorenstraße 5, 40225 Düsseldorf, Germany; 6 Institute of Pharmaceutical Biology and Biotechnology, Faculty of Mathematics and Natural Sciences, Heinrich Heine University, Universitätsstraße 1, 40225 Düsseldorf, Germany; 7 Department of Hematology, Oncology and Clinical Immunology, Medical Faculty and University Hospital Düsseldorf, Moorenstraße 5, 40225 Düsseldorf, Germany

**Keywords:** Cancer metabolism, Small molecules, Cancer metabolism, Drug development, Mechanism of action

## Abstract

Inhibition of the mitochondrial metabolism offers a promising therapeutic approach for the treatment of cancer. Here, we identify the mycotoxin viriditoxin (VDT), derived from the endophytic fungus *Cladosporium cladosporioides*, as an interesting candidate for leukemia and lymphoma treatment. VDT displayed a high cytotoxic potential and rapid kinetics of caspase activation in Jurkat leukemia and Ramos lymphoma cells in contrast to solid tumor cells that were affected to a much lesser extent. Most remarkably, human hematopoietic stem and progenitor cells and peripheral blood mononuclear cells derived from healthy donors were profoundly resilient to VDT-induced cytotoxicity. Likewise, the colony-forming capacity was affected only at very high concentrations, which provides a therapeutic window for cancer treatment. Intriguingly, VDT could directly activate the mitochondrial apoptosis pathway in leukemia cells in the presence of antiapoptotic Bcl-2 proteins. The mitochondrial toxicity of VDT was further confirmed by inhibition of mitochondrial respiration, breakdown of the mitochondrial membrane potential (ΔΨm), the release of mitochondrial cytochrome c, generation of reactive oxygen species (ROS), processing of the dynamin-like GTPase OPA1 and subsequent fission of mitochondria. Thus, VDT-mediated targeting of mitochondrial oxidative phosphorylation (OXPHOS) might represent a promising therapeutic approach for the treatment of leukemia and lymphoma without affecting hematopoietic stem and progenitor cells.

## Introduction

Targeting mitochondrial metabolism represents a novel approach for cancer treatment. In contrast to previous assumptions, mitochondria are considered to be the central players in tumor development and therapy resistance. Originally, Otto Warburg observed a high rate of glycolysis followed by lactic acid fermentation in tumor cells even in the presence of abundant oxygen (aerobic glycolysis; also known as “Warburg-effect”). Consequently, he postulated that tumor cells comprise dysfunctional mitochondria which are causative for enhanced glycolysis and tumorigenesis (“Warburg hypothesis”) [1, 2]. Meanwhile however, it has been disclosed that tumor cells do not prefer the ATP-inefficient route of glycolysis in order to compensate for a dysfunctional oxidative phosphorylation (OXPHOS) pathway in damaged mitochondria. Instead, they rather utilize the increased glucose consumption for anabolic processes required for tumor progression [1–3]. Moreover, it has been shown that tumor cells are equipped with functional mitochondria, which are required for tumor development [4]. Especially, leukemic cells and cancer stem cells display a high mitochondrial mass and even solid tumors show substantial metabolic flexibility by switching to mitochondrial OXPHOS in order to acquire therapy resistance to conventional genotoxic therapies as well as targeted therapies [4, 5]. Consequently, targeting mitochondrial metabolism has become a promising approach to overcome therapy resistance of hematological cancers and solid tumors. These approaches include drugs that target the electron transport chain (ETC) and OXPHOS, the tricarboxylic acid (TCA) cycle, or mitochondrial translation [4, 6].

To identify novel anticancer drugs, we screened a library of natural products [7] and identified the mycotoxin viriditoxin (VDT). VDT has been isolated from different fungal species including *Paecilomyces variotii, Cladosporium cladosporioides*, and *Aspergillus fumigatus*, which can infect organisms as diverse as jellyfishes (*Nemopilema nomurai*) or flowering plants *(Lawsonia alba)* [8, 9]. VDT exhibits broad-spectrum antibacterial activity against Gram-positive bacteria via inhibition of the bacterial protein FtsZ, which is a close structural homolog of eukaryotic tubulin and is involved in bacterial cell division [10, 11]. Moreover, Kundu et al. reported that VDT induces apoptosis, mitotic catastrophe, G_2_/M phase cell cycle arrest and autophagic cell death in human prostate cancer cells. However, so far VDT has mainly been investigated in various solid tumor cell lines where it displayed only a moderate cytotoxic potential [8, 12, 13].

Here, we demonstrate that VDT potently induced apoptosis in rapid kinetics with low IC_50_ values in the human T cell leukemia cell line Jurkat (as well as in other leukemic cell lines such as HL60, HPALL, K562, KOPTK1, MOLT4, SUPB15) and the human B cell lymphoma cell line Ramos, whereas all solid tumor cell lines tested (143B, HCT116, HeLa, HT29. MCF7, RT112, SH-SY5Y) were much more resilient. Moreover, non-transformed cells like human hematopoietic stem and progenitor cells (HSPC) and peripheral blood mononuclear cells (PBMC) were mostly unaffected thus providing a therapeutic window. Since tumor cells frequently inactivate the mitochondrial apoptosis pathway by overexpression of antiapoptotic Bcl-2 members (such as Bcl-2, Bcl-xL, or Mcl-1) to acquire therapy resistance—it was remarkable that VDT was proficient to induce apoptosis in Bcl-2 overexpressing Jurkat cells or in Bax-/Bak-deficient DG75 cells. Thus, VDT was able to directly activate the mitochondrial apoptosis pathway as documented by the breakdown of mitochondrial respiration (OXPHOS) and ΔΨm, the release of mitochondrial cytochrome c, ROS production and subsequent mitochondrial fragmentation. Therefore, targeting mitochondrial OXPHOS via VDT might represent a promising therapeutic approach for the treatment of leukemia and lymphoma without hematotoxic side effects.

## Results

### VDT is highly cytotoxic in leukemia and lymphoma cells compared to solid tumor cells

**Fig. 1:**
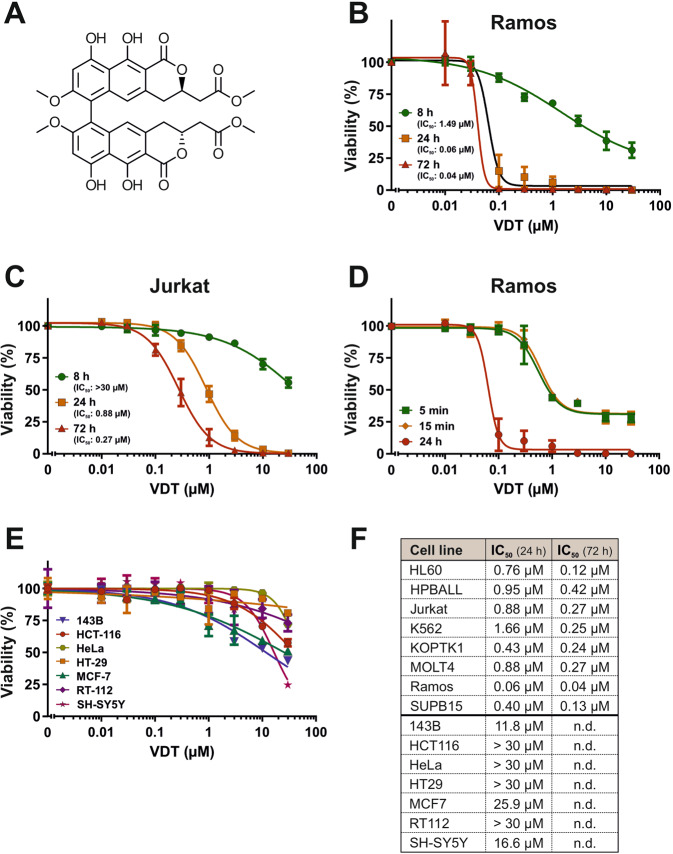
VDT is highly cytotoxic in leukemia and lymphoma cells in comparison to solid tumor cells. **A** Structure of viriditoxin (VDT). **B**, **C** Cytotoxicity in Ramos (**B**; human B cell lymphoma) or Jurkat cells (**C**; human T cell acute lymphoblastic leukemia; T-ALL) was determined after 24 h or 72 h treatment with VDT using the AlamarBlue® viability assay. The respective IC_50_ values are given in parenthesis. **D** Ramos cells were treated with increasing VDT concentrations for 5 min, 15 min, or 24 h. In the first two treatment series, VDT was washed away at the end of the indicated incubation periods (5 or 15 min) and cells were further cultivated in medium. After 24 h upon start of the experiment, viability was determined by MTT assay. **E** Cytotoxicity of VDT after 24 h of incubation was determined by MTT assay in a panel of human solid tumor cell lines including 143B (osteosarcoma), HCT116 (colorectal carcinoma), HeLa (cervix carcinoma), HT29 (colon carcinoma), MCF7 (breast carcinoma), RT112 (urinary bladder carcinoma) and SH-SY5Y (neuroblastoma). **F** Overview of the resulting IC_50_ values upon VDT treatment of the individual tested cell lines after 24 h and 72 h. Upper panel: leukemia and lymphoma cell lines. In addition, to Jurkat and Ramos cells IC_50_ values of 6 additional human leukemic cell lines i.e., HL60 (acute myeloid leukemia; AML), HPBALL (T cell acute lymphoblastic leukemia; T-ALL), K562 (chronic myeloid leukemia; CML), KOPTK1 (T-ALL), MOLT4 (T-ALL), SUPB15 (B cell acute lymphoblastic leukemia; B-ALL) are shown. Viability assays for these leukemic cell lines are depicted in Supplementary Fig. 1. Lower panel: solid tumor cell lines. “n.d.” indicates “not done”.

To discover novel anticancer drugs, we screened a library of 300 different natural products [7] for their cytotoxic potential in Ramos (human B cell Burkitt lymphoma) and Jurkat (human T cell acute lymphoblastic leukemia; T-ALL) cells. VDT, characterized by its 6,6’-binaphthopyranone structure (Fig. 1A), was isolated from the fungus *Cladosporium cladosporioides* [9] and was extremely toxic, especially in Ramos but also in Jurkat cells (Fig. 1B, C). The resulting IC_50_ values after a 24-hour incubation were 0.06 µM for Ramos cells and 0.88 µM for Jurkat cells (Fig. 1B, C, F). In addition, we treated Ramos cells for 5 min or 15 min with VDT. Subsequently, cells were washed and further incubated for 24 h. Thereby, we could show that the exposure of 5 min to VDT was sufficient to cause severe and irreversible cytotoxicity (Fig. 1D). Finally, we tested VDT on a panel of solid tumor cell lines (143B, HCT116, HeLa, HT29, MCF7, RT112, SH-SY5Y). The resulting IC_50_ values of all solid tumor cell lines after 24 h VDT-treatment were at least 10 times higher than in Ramos or Jurkat cells. For the majority of the solid tumor cell lines used, significant cytotoxicity was observed only at high concentrations above 10 µM (Fig. 1E, F). To corroborate that VDT targets primarily leukemia and lymphoma cells we tested 6 additional human leukemia cell lines i.e., HL60 (acute myeloid leukemia; AML), HPBALL (T cell acute lymphoblastic leukemia; T-ALL), K562 (chronic myeloid leukemia; CML), KOPTK1 (T-ALL), MOLT4 (T-ALL), and SUPB15 (B cell acute lymphoblastic leukemia; B-ALL). Thus, we could demonstrate that VDT displays a high cytotoxic potential predominantly in human lymphoma and leukemia cells whereas solid tumor cells were obviously less sensitive (Fig. 1F, Supplementary Fig. 1).

### VDT is a potent inducer of apoptosis in leukemia and lymphoma cells with rapid kinetics

**Fig. 2:**
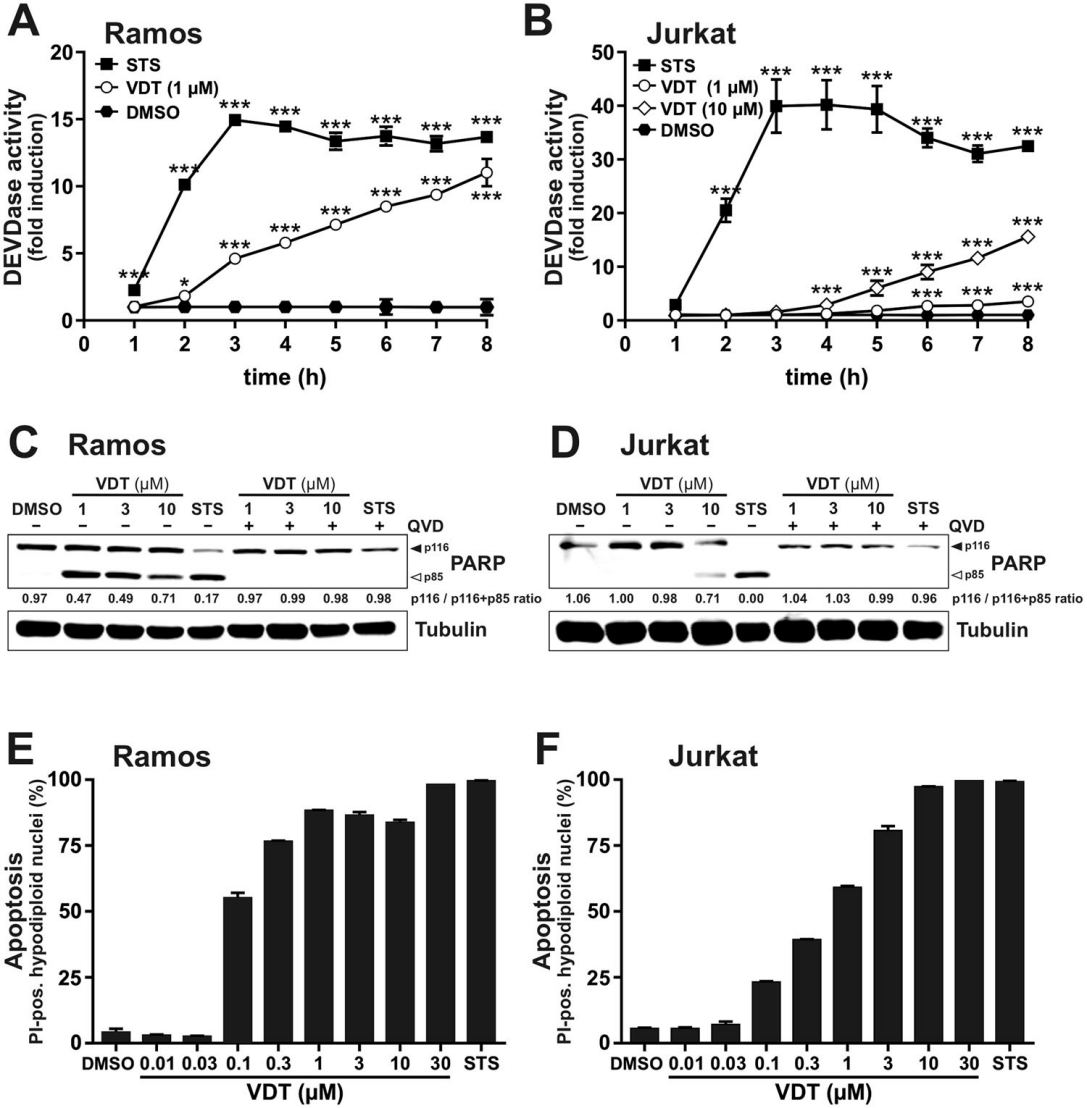
VDT induces apoptosis in rapid kinetics in leukemia and lymphoma cells. (**A**) Ramos or (**B**) Jurkat cells were treated with VDT or 2.5 µM staurosporine (STS; as a positive control for apoptosis induction) for up to 8 h. Subsequently, DEVDase activity as a surrogate marker for caspase-3 activation was determined via measurement of the fluorescence of the profluorescent caspase-3 substrate DEVD-AMC in a spectrofluorometer. The slope of the linear range of fluorescence increase served as a measure for DEVDase activity. The DMSO control values were set to 1 and the normalized relative fold induction was calculated as described in Materials and Methods. Error bars = SD of three independent experiments performed in triplicates; *p*-values were calculated by two-way ANOVA with the Holm–Sidak post-test; *p ≤ 0.05, ***p ≤ 0.001. (**C**, **D**) Cleavage of the caspase-3 substrate poly(ADP-ribose) polymerase 1 (PARP) as an indicator for apoptotic cell death was measured in (**C**) Ramos and (**D**) Jurkat cells after 8 h of incubation via immunoblotting. Cells were treated with the indicated concentrations of VDT (µM) or 2.5 µM STS either alone or in combination with the pan-caspase inhibitor QVD (10 µM). Solid arrowheads indicate the uncleaved p116 form of PARP; open arrowheads indicate the cleaved p85 form. Immunoblotting for tubulin was used as loading control. Numbers under PARP immunoblot indicate densitometric analyses of the ratio of unfragmented (p116) to total PARP (p116/p116 + p85). **E**, **F** Apoptosis-related DNA degradation was detected after 24 h of incubation via flow-cytometric measurement of propidium iodide stained apoptotic hypodiploid nuclei in (**E**) Ramos or (**F**) Jurkat cells.

To investigate whether the observed induction of cell death is due to apoptosis, we used different readouts for the detection of apoptosis. To measure the catalytic caspase activity, we used the profluorescent caspase-3 substrate Ac-DEVD-AMC to monitor the activity of caspase-3 upon treatment with VDT. The measurement of the 8-hour kinetics revealed a rapid activation of caspase-3 already after 3–5 h in Ramos and Jurkat cells (Fig. 2A, B). Similar to the cytotoxicity measurements, caspase activation in Jurkat cells required a higher concentration of VDT (10 µM) than in Ramos cells (Fig. 2A, B). However, VDT-induced caspase activation was not as rapid and pronounced as upon treatment with the potent apoptotic stimulus staurosporine (STS) that was used as positive control [14]. In addition, we observed in both cell lines a significant cleavage of the caspase substrate poly(ADP-ribose) polymerase-1 (PARP) upon 8 h VDT treatment. This cleavage was prevented by co-treatment with the pan-caspase inhibitor QVD (Fig. 2C, D). VDT also induced a pronounced and dose-dependent increase in apoptotic hypodiploid nuclei in both cell lines (Fig. 2E, F).

### VDT displays low cytotoxicity in non-transformed HSPC and PBMC and offers a therapeutic window

**Fig. 3:**
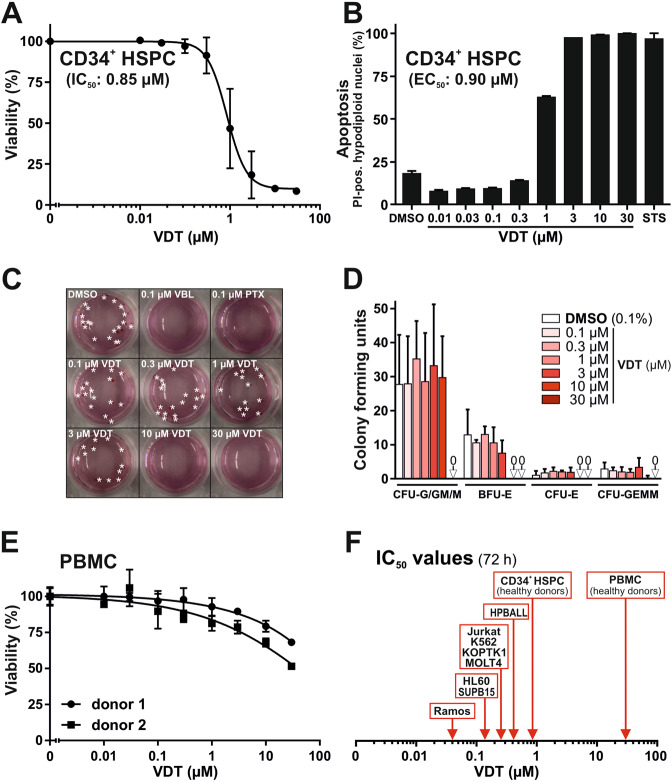
VDT shows low cytotoxicity in non-transformed HSPC and PBMC and displays a therapeutic window. **A** Cytotoxicity in hematopoietic stem and progenitor cells (HSPC) derived from healthy donors via apheresis and magnetic-activated cell sorting (MACS) against the stem cell marker CD34 was determined after 72 h of incubation with VDT using MTT viability assay. **B** HSPC from healthy donors were treated for 24 h with either increasing concentrations of VDT or 2.5 µM staurosporine (STS). Subsequently, apoptotic events were identified via flow-cytometric measurement of apoptotic hypodiploid nuclei. **C** HSPC were plated at low cell density in semi-solid medium MethoCult 4436 and treated with 0.1% (v/v) DMSO, viriditoxin (VDT), paclitaxel (PTX; 0.1 µM) or vinblastine (VBL; 0.1 µM) for 14 d. Thereafter, the resulting colonies were counted and differentiated under light microscope. Depicted are representative pictures. Asterisks indicate BFU-E and CFU-E colonies. **D** To determine the proliferation colonies were counted under light microscope after 14 days of the colony-forming unit (CFU) assay: (i) colony-forming unit-granulocyte, colony-forming unit-granulocyte/macrophage, colony-forming unit-macrophage (CFU-G/GM/M), (ii) colony-forming unit-erythroid (CFU-E), (iii) burst-forming unit-erythroid (BFU-E), and (iv) colony-forming unit-granulocyte/erythrocyte/macrophage/megakaryocyte (CFU-GEMM). **E** Cytotoxicity in human peripheral blood mononuclear cells (PBMC) obtained from two healthy donors was determined after 72 h of incubation with VDT using MTT viability assay. **F** Overview of the determined IC_50_ values for 72 h of incubation with VDT in human transformed cell lines (Ramos, Jurkat, HL60 (AML), HPBALL (T-ALL), K562 (CML), KOPTK1 (T-ALL), MOLT4 (T-ALL), and SUPB15 (B-ALL); for IC_50_ values see Fig. 1F and Supplementary Fig. 1) and untransformed cells (CD34^+^ HSPC IC_50_: 0.85 µM; PBMC IC_50_: > 30 µM).

In order to evaluate the hematotoxic effects of VDT, we used CD34-positive human hematopoietic stem and progenitor cells (HSPC) from healthy donors. After 72 h of treatment with VDT, its cytotoxic effect on HSPC was significantly reduced as reflected by a 21-fold greater IC_50_ of 0.85 µM (Fig. 3A) in comparison to an IC_50_ of 0.04 µM for Ramos cells (Fig. 1B, F) and still 3-fold in comparison to Jurkat cells (IC_50_: 0.27 µM; Fig. 1C, F). Similar results were obtained for HSPC when apoptosis was measured by the amount of apoptotic hypodiploid nuclei after 24 h (EC_50_: 0.9 µM; Fig. 3B). Next, we determined the effect of VDT on the proliferation potential of HSPC in colony-forming assays. Even at high concentrations of VDT (up to 3 µM) HSPC did not show any reduced proliferation or differentiation (Fig. 3C, D). In contrast, the anticancer drugs vinblastine and paclitaxel (which are applied in leukemia and lymphoma therapy) completely inhibited proliferation and differentiation already at very low concentrations (Fig. 3C). We additionally tested the cytotoxicity of VDT on human peripheral blood mononuclear cells (PBMC) isolated from healthy donors. Yet, VDT was only slightly toxic in PBMC (IC_50_ > 30 µM; Fig. 3E). Thus, VDT offers a therapeutic window for the treatment of leukemia and lymphoma (Fig. 3F).

### The cytotoxic potential of VDT is independent of tubulin polymerization

VDT is known to exhibit a broad-spectrum antibacterial activity based on the inhibition of the bacterial protein FtsZ, whose three-dimensional structure is closely related to eukaryotic tubulin [10]. Furthermore, Kundu et al. reported that VDT induces mitotic catastrophe and autophagic cell death in prostate cancer cells presumably by disrupting tubulin polymerization [8]. Similarly, Su et al. reported that VDT displays an antimitotic activity in the ovarian cancer cell line SK-OV-3 by stabilization of microtubule polymers [13]. Based on these findings, we investigated whether the cytotoxic mechanism of VDT might be attributed to disturbed tubulin polymerization. However, using a tubulin polymerization assay we detected no significant influence on the polymerization rate of tubulin (Supplementary Fig. 2A, B). There was also no accumulation of polymerized tubulin (Supplementary Fig. 2C) nor induction of cell cycle arrest which would be characteristic for an antimitotic toxin (Supplementary Fig. 2D). This discrepancy is most likely attributed to the fact that Su et al. observed an effect on tubulin polymerization only at high VDT concentrations (50 µM and 100 µM) whereas—as in our case – they did not observe any effect at 10 µM of VDT [13]. Likewise, they observed an effect on cell cycle arrest at rather high VDT concentrations (>20 µM). Therefore, we excluded a disturbed tubulin polymerization as the major mechanism for VDT-induced cytotoxicity.

### VDT activates the mitochondrial apoptosis pathway in the presence of Bcl-2 and in Bax-/Bak-deficient cells

Next, we investigated which apoptotic signaling pathway was affected by VDT. There exist at least two major pathways leading to the apoptotic demise of the cell – the extrinsic death receptor pathway and the intrinsic mitochondrial apoptosis pathway. In death receptor-mediated apoptosis, the stimulation of receptors (such as CD95/Apo-1/Fas, TRAIL-R1, or TRAIL-R2) leads to the activation of the initiator caspase-8 that proteolytically activates the effector caspase-3. The intrinsic mitochondrial cytochrome c/Apaf-1 pathway is usually activated by cell stress such as DNA damage inflicted during radio- and chemotherapy and is initiated by the release of cytochrome c from the mitochondria. The mitochondrial cytochrome c release is mediated by proapoptotic Bcl-2 proteins (such as Bax or Bak). Anti-apoptotic members of the Bcl-2 family (such as Bcl-2, Bcl-xL, Mcl-1) can in turn inhibit the release of cytochrome c, thereby blocking the mitochondrial apoptosis pathway [15]. In the cytosol, cytochrome c subsequently activates the initiator caspase-9 via the adapter protein Apaf-1 in a high molecular signal complex termed apoptosome. Activated caspase-9 then cleaves and activates effector caspases-3 and -7 [16].

Since caspase-8 represents the central initiator caspase of the death receptor pathway we used caspase-8 deficient Jurkat cells to investigate whether VDT-induced apoptosis is mediated via the extrinsic apoptosis pathway. As shown in Supplementary Fig. 3, VDT induced apoptosis and cleavage of the caspase substrate PARP in both, parental caspase-8 proficient Jurkat cells as well as in caspase-8 deficient Jurkat cells, whereas death receptor stimulation via TRAIL was blocked when caspase-8 was not present. Thus, the involvement of the death receptor pathway in VDT-induced apoptosis could be excluded.

**Fig. 4:**
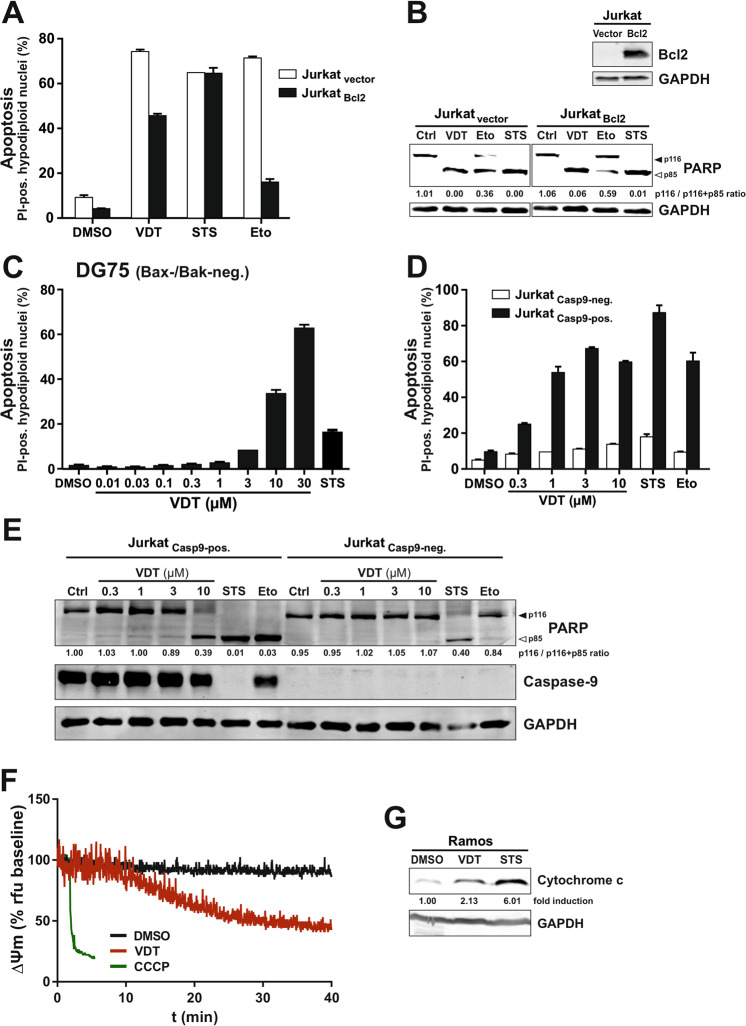
VDT directly activates the mitochondrial apoptosis pathway in Bcl-2 overexpressing Jurkat cells or Bax-/Bak-deficient DG75 cells. **A** VDT induces apoptosis in the presence of antiapoptotic Bcl-2. Apoptosis induction was analyzed in Jurkat cells stably transfected with vectors encoding Bcl-2 (Jurkat Bcl2; black bars) or empty vector (Jurkat vector; white bars). 5 × 10^4^ cells were stimulated with viriditoxin (VDT; 10 µM), staurosporine (STS; 2.5 µM), etoposide (Eto; 50 µM) or diluent control (DMSO; 0.1% v/v). After 24 h, apoptosis was assessed by propidium iodide staining of apoptotic hypodiploid nuclei and flow cytometry. **B** Lower panel: 2 × 10^6^ Jurkat cells transfected with empty vector (Jurkat-vector) or Bcl-2 (Jurkat-Bcl2; expression level of Bcl-2 is shown in upper panel) were treated with viriditoxin (VDT; 10 µM), staurosporine (STS; 2.5 µM), etoposide (Eto; 50 µM) or DMSO diluent control (Ctrl; 0.1% v/v). After 8 h, cellular proteins were resolved by SDS-PAGE and investigated for the proteolytic processing of PARP by immunoblotting. Solid arrowheads indicate the uncleaved form of PARP (p116); open arrowheads indicate the cleaved form (p85). Immunoblotting for glyceraldehyde 3-phosphate dehydrogenase (GAPDH) was used as loading control. Numbers under PARP immunoblot indicate densitometric analyses of the ratio of unfragmented (p116) to total PARP (p116/p116 + p85). **C** VDT induces apoptosis in Bax- and Bak-deficient DG75 Burkitt lymphoma cells. 5 × 10^4^ DG75 cells were treated for 24 h with the indicated concentrations of VDT, staurosporine (STS; 2.5 µM), or diluent control (DMSO; 0.1% v/v), respectively. Subsequently, apoptosis was assessed by propidium iodide staining of apoptotic hypodiploid nuclei and flow cytometry. **D**, **E** Caspase-9 is required for VDT-induced apoptosis. **D** 5 × 10^4^ caspase-9 deficient Jurkat cells transfected with empty vector (Jurkat Casp9-neg.) or untagged wild type caspase-9 (Jurkat Casp9-pos.) were treated for 24 h with the indicated concentrations of VDT, staurosporine (STS; 2.5 µM), etoposide (Eto; 50 µM) or diluent control (DMSO; 0.1% v/v), respectively. Subsequently, apoptosis was assessed by propidium iodide staining of apoptotic hypodiploid nuclei and flow cytometry. **E** Caspase-9 deficient (Jurkat Casp9-neg.) or capase-9 proficient Jurkat cells (Jurkat Casp9-pos.) were treated with the indicated concentrations of VDT, staurosporine (STS; 2.5 µM), etoposide (Eto; 50 µM) or DMSO diluent control (Ctrl; 0.1% v/v). After 8 h, the proteolytic processing of PARP and caspase-9 was detected by immunoblotting. Solid arrowheads indicate the uncleaved form of PARP (p116); open arrowheads indicate the cleaved form (p85). Immunoblotting for glyceraldehyde 3-phosphate dehydrogenase (GAPDH) was used as loading control. Numbers under PARP immunoblot indicate densitometric analyses of the ratio of unfragmented (p116) to total PARP (p116/p116 + p85). **F** Monitoring of the mitochondrial membrane potential (ΔΨm) of Ramos cells after addition of 10 µM VDT, 0.1% (v/v) DMSO (negative control), or 10 µM CCCP (mitochondrial uncoupler, positive control) by flow cytometric measurement of TMRE fluorescence. **G** For the detection of mitochondrial release of cytochrome c, Ramos cells (5 × 10^6^) were treated with 0.1% DMSO (0.1% v/v), 1 µM VDT, or 2.5 µM STS for 8 h. Subsequently, cytosolic extracts were prepared by applying a digitonin lysis buffer protocol. Cytosolic cytochrome c was detected by immunoblotting. Numbers under cytochrome c immunoblot indicate densitometric analyses of the fold detection of cytochrome c relative to loading control (GAPDH).

Activation of the mitochondrial apoptosis pathway can be blocked by overexpression of anti-apoptotic Bcl-2 proteins (such as Bcl-2, Bcl-xL, or Mcl-1). Intriguingly, VDT mediated apoptosis induction and PARP-cleavage was only attenuated but not blocked in Jurkat cells overexpressing Bcl-2 (Fig. 4A, B). As to be expected, the apoptosis induction by the DNA-damaging anticancer drug etoposide was prevented in the presence of Bcl-2 (Fig. 4A, B). Since staurosporine is proficient to induce apoptosis in Bcl-2 or Bcl-xL overexpressing cells [14] apoptosis induction was not affected by Bcl-2 (Fig. 4A, B). In addition, we could show that VDT was proficient to induce apoptosis in the human B cell Burkitt lymphoma cell line DG75 that has been shown to be Bax- and Bak-deficient [17, 18] (Fig. 4C). However, when we used caspase-9 deficient Jurkat cells VDT-induced apoptosis, and PARP-cleavage was completely blocked (Fig. 4D, E). Thus, these data indicate that VDT can activate the mitochondrial apoptosis pathway in a direct way that could not be blocked by Bcl-2 overexpression or Bax-/Bak-deficiency.

### VDT impairs mitochondrial structure and function

In the following, we focused on mitochondria as the executioners of the intrinsic apoptosis pathway. First, we measured the effect of VDT on the mitochondrial membrane potential (ΔΨm). As shown in Fig. 4F, VDT induced a substantial breakdown of the ΔΨm within 30 min—however not as fast as the protonophore CCCP that was used as positive control. In addition, VDT induced the mitochondrial release of cytochrome c (Fig. 4G).

**Fig. 5:**
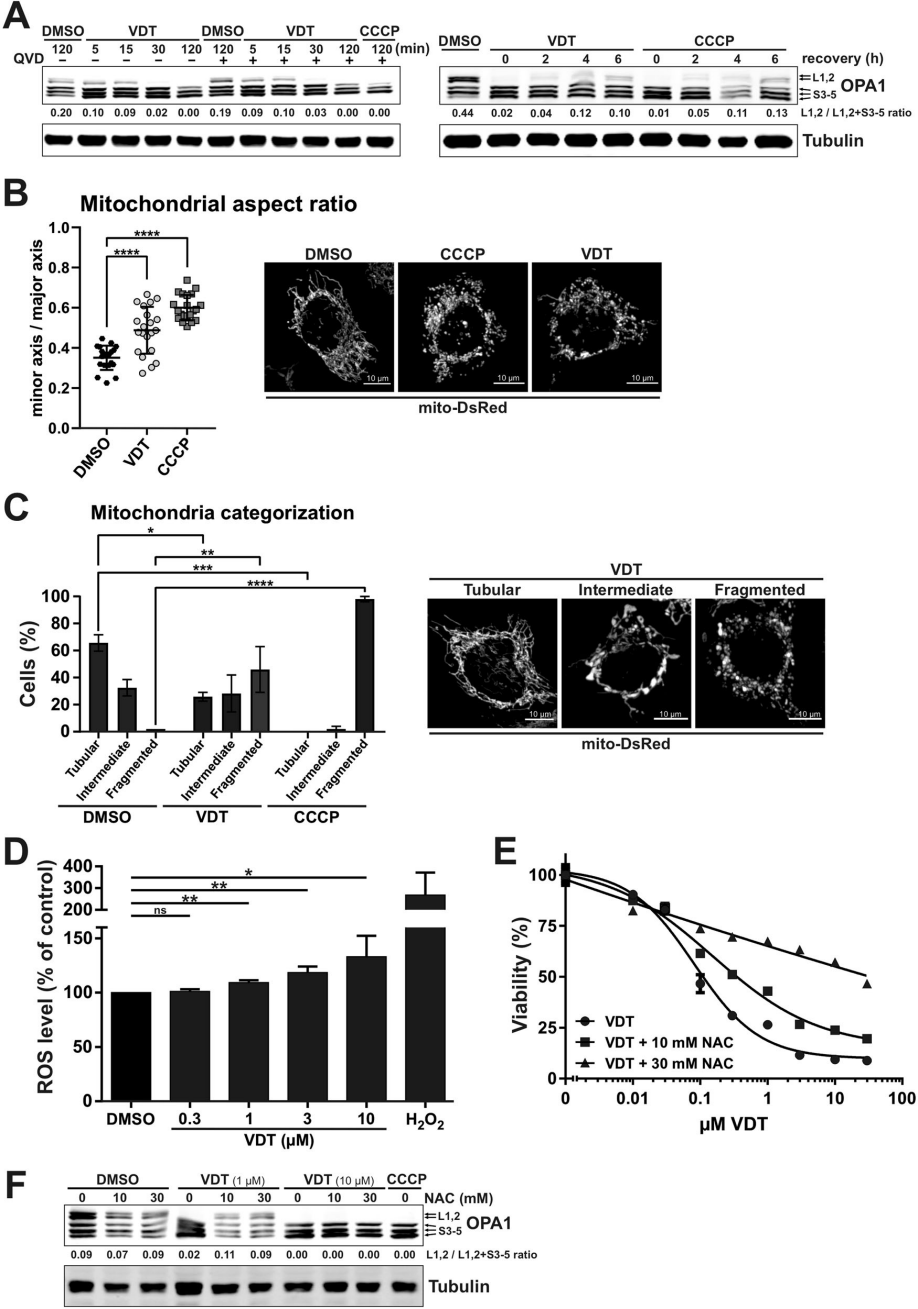
VDT impairs mitochondrial structure and function. **A** Left panel: The kinetics of VDT-induced OPA1 cleavage as determined by immunoblotting in Ramos cells. Co-treatment with the pan-caspase-inhibitor QVD (10 µM) was conducted to ensure independence from apoptotic signaling. Treatment with CCCP (10 µM) served as a positive control. Right panel: To study the recovery of long forms of OPA1, Ramos cells were treated for 30 min with either VDT (10 µM) or CCCP (10 µM), followed by substance removal and a recovery time of up to 6 h. Numbers under OPA1 immunoblot indicate densitometric analyses of the ratio of the long forms of OPA1 (L1, L2) to total OPA1 (L1,L2/L1,L2 + S3-5). **B**, **C** Changes in mitochondrial morphology after treatment with DMSO (0.1% v/v), VDT (10 µM), or CCCP (10 µM; positive control) for 7 h were assessed by spinning disc confocal microscopy of HeLa cells stably expressing the fluorescent dye mito-DsRed, which stains the mitochondrial matrix. **B** Shown are representative images. For mitochondrial aspect ratio determination, the minor axis was divided by the major axis and manually measured for 30 mitochondria of at least 20 cells per condition averaged for each cell, with individual values and mean shown. Error bars show SD, statistics: one-way ANOVA with Dunnett’s multiple comparison test. ****p ≤ 0.0001. **C** Mitochondrial morphology was assessed by categorizing 40 to 60 cells per condition in 2 independent experiments into tubular, intermediate, or fragmented (representative microscopy images of VDT-treated cells are shown next to the graph). The bars show the average, error bars show the range; statistics: two-way ANOVA with Dunnett’s multiple comparison test. ****p ≤ 0.0001. **D** VDT-induced effects on intracellular reactive oxygen species (ROS) activity as analyzed via DCF-assay. Ramos cells were loaded with the fluorogenic dye 2’,7’-dichlorodihydrofluorescein diacetate (H_2_DCF-DA; 20 µM) and then treated with the indicated concentrations of VDT or 1 mM H_2_O_2_ (positive control) for 6 h. Subsequently, the ROS mediated generation of fluorescent DCF was measured in a spectrophotometer. Endogenous ROS level of cells treated with DMSO (0.1% v/v) was set to 100%. Error bars = SD of three independent experiments performed in triplicates; *p*-values were calculated by one sample *t*-test; *p ≤ 0.05, **p ≤ 0.01. **E** Measurement of the cytotoxicity of VDT with or without co-treatment with the ROS scavenger N-acetylcysteine (NAC; 10 or 30 mM) after an incubation period of 24 h via resazurin reduction assay (AlamarBlue® assay). **F** Immunoblot of Ramos cells treated for 60 min with VDT (1 or 10 µM) with or without NAC pretreatment (10 or 30 mM). CCCP served as positive control for OPA1 cleavage. Numbers under OPA1 immunoblot indicate densitometric analyses of the ratio of the long forms of OPA1 (L1, L2) to total OPA1 (L1,L2 / L1,L2 + S3-5).

Another hallmark of the mitochondrial apoptosis pathway is the fragmentation (fission) of mitochondria. In this context, the dynamin-like GTPase OPA1 plays a major role. OPA1 is a central regulator in the maintenance of mitochondrial homeostasis, serves as a sensor of mitochondrial stress such as impaired mitochondrial membrane potential and interconnects mitochondrial quality control and intrinsic apoptosis. The activity of OPA1 is regulated via proteolytic processing through OMA1 and YME1L1 and balances the fusion and fission of the mitochondrial network. Stress-induced cleavage of OPA1 and the associated loss of its long isoforms (L-OPA1) shifts the balance towards increased fission, leading to mitochondrial fragmentation and increased sensitivity to proapoptotic stimuli [19, 20]. Consequently, we investigated the impact of VDT on OPA1 and observed that VDT caused a rapid cleavage of OPA1 within several minutes, which was not affected by caspase-inhibition via QVD (Fig. 5A; left panel). The VDT-induced loss of long OPA1 isoforms was reversible as reflected by their reappearance within 6 h upon VDT-removal (Fig. 5A; right panel). In addition, we observed that the loss of long OPA1 isoforms (L-OPA1) was accompanied by substantial fragmentation (fission) of the mitochondrial network after 7 h (Fig. 5B, C). Since severe mitochondrial damage and oxidative stress are often mutually dependent, we subsequently investigated the effect of VDT on cellular reactive oxygen species (ROS) generation. Measurements with the fluorescent dye H_2_-DCF-DA revealed that VDT distinctly increased the ROS level compared to the DMSO-treated control, indicating the induction of oxidative stress (Fig. 5D). To determine whether the observed oxidative stress was responsible for the measured cytotoxicity, we analyzed in how far the antioxidant *N*-acetylcysteine (NAC) could attenuate VDT-induced cell death. Thereby, we observed that co-treatment with NAC reduced toxicity, suggesting that oxidative stress is at least partially responsible for VDT’s cytotoxicity (Fig. 5E). Furthermore, we could show that co-treatment with NAC also prevented the processing of the long isoforms of OPA1 upon treatment with 1 µM VDT (Fig. 5F).

### VDT impairs mitochondrial respiration

**Fig. 6:**
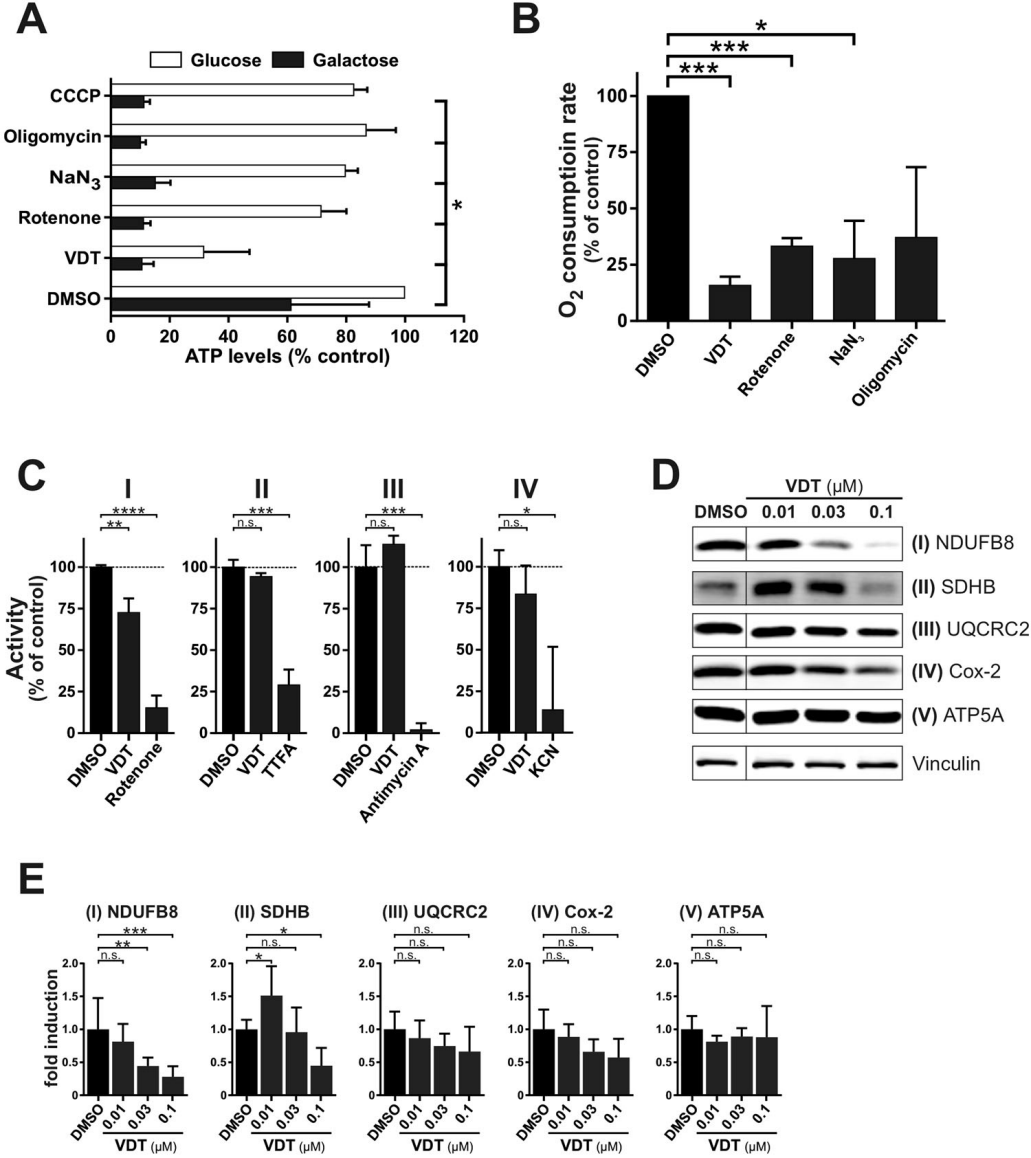
VDT impairs mitochondrial respiration. **A** Measurement of the effect of VDT (10 µM) and a selection of known mitotoxins on the ATP levels of Ramos cells. Ramos cells were treated for 90 min with the indicated agents in full growth medium containing either glucose or galactose as the only available sugar. Galactose alone forces the cells to rely entirely on OXPHOS for ATP synthesis. The following complex-specific inhibitors of the respiratory chain were used: rotenone (complex I; 10 µM), sodium azide (NaN_3_, 1 mM; complex IV), oligomycin A (complex V; 10 µM). Subsequently, the ATP levels were measured using the luminescence-based mitochondrial ToxGlo™ assay (Promega). The depicted values were normalized to cells treated with DMSO (0.1% v/v) in glucose containing growth medium (set to 100%). Error bars = SD of three independent experiments performed in triplicates; *p*-values were calculated by two-way ANOVA with the Holm–Sidak post-test; *p ≤ 0.05. **B** Comparative measurement of the oxygen consumption rate of Ramos cells treated with VDT (10 µM) or a range of known complex-specific inhibitors of the respiratory chain (see Fig. 6A) using the MITO-ID® Extracellular O_2_ Sensor Kit (High Sensitivity; Enzo). The oxygen consumption rate of cells treated with 0.1% DMSO (v/v) was set to 100%. Error bars = SD of three independent experiments performed in triplicates; *p*-values were calculated by one sample *t*-test; *p ≤ 0.05, ***p ≤ 0.001. **C** The activities of the individual complexes of the respiratory chain were measured after treatment with VDT (10 µM) or respective complex inhibitors [complex I: 10 µM rotenone; complex II: 10 mM thenoyltrifluoroacetone (TTFA); complex III: 10 µM antimycin A; complex IV: 1 mM potassium cyanide (KCN)] for 15 min using the corresponding MitoCheck® kit (Cayman Chemical; utilizing mitochondria isolated from bovine heart). Depicted activities were normalized to cells treated with DMSO (0.1% v/v). *p*-values were calculated by one sample *t*-test; *p ≤ 0.05, ***p ≤ 0.001, ns not significant. **D** For each complex of the respiratory chain a subunit was selected which is unstable and degraded if the respective complex is incorrectly assembled. The following complex-specific proteins were detected by immunoblotting: NADH ubiquinone oxidoreductase subunit B8 (NDUFB8; complex I), succinate dehydrogenase subunit B (SDHB; complex II), ubiquinol-cytochrome c reductase core protein 2 (UQCRC2; complex III), cytochrome c oxidase-2 (Cox-2; complex IV), ATP synthase F1α (ATP5A; complex V). Therefore, Ramos cells were treated with nanomolar concentrations of VDT for 24 h before an immunoblot against these subunits was performed. Vinculin served as loading control. **E** The bar chart shows the quantification of the signal of immunoblots from Fig. 6D for the subunits of ETC complexes I - V, based on at least three independent experiments. Error bars = SD of at least three independent experiments; *p*-values were calculated by two-way ANOVA with the Holm–Sidak post-test; *p ≤ 0.05, **p ≤ 0.01, ***p ≤ 0.001, ns not significant.

Since a major function of the mitochondria is the supply of cellular ATP, we investigated how far VDT affects the cellular ATP level. To separate potential effects on glycolysis from oxidative phosphorylation, we provided the cells with either glucose or galactose as the only sugar supply within the medium. The glycolytic degradation of galactose instead of glucose does not lead to a net ATP gain. This forces the cell to rely entirely on OXPHOS for energy production which makes it particularly sensitive to disrupters of the respiratory chain [21]. Under galactose conditions, VDT caused a drastic and rapid drop in cellular ATP levels, similar to a series of electron transport chain inhibitors used as control (Fig. 6A). This suggests that VDT inhibits the mitochondrial respiration which could be corroborated by demonstrating that VDT caused a rapid and significant decrease in oxygen consumption rate (Fig. 6B). To determine whether VDT might directly inhibit one of the four electron transport chain (ETC) complexes, we measured the activity of each complex in purified mitochondria under VDT treatment. Since ETC complexes are usually degraded upon incorrect assembly, we performed immunoblot analyses to monitor the degradation of specific subunits of the individual complexes after 24 h. Thus, we could observe a decreased activity upon treatment with VDT for complex I – whereas the decreased activity of complex IV was not significant (Fig. 6C). The reduced activity level of complex I was accompanied by a decreased level of the corresponding subunit (NDUFB8) in the immunoblot (Fig. 6D, E). We also observed a significant reduction in the expression of the subunit SDHB of complex II (Fig. 6D, E) which however, did not correlate with a decreased activity (Fig. 6C). For complex IV only a slight and not significant decrease in the expression of the corresponding subunit Cox-2 could be observed (Fig. 6D, E).

### Effect of VDT on mitochondrial ribosomal proteins and mitochondrial translation

In order to identify potential targets of VDT, we performed an unbiased proteomic approach using thermal proteome profiling (TPP). This method enables the detection of drug-protein interactions in living cells by identification of proteins whose thermal stabilities are – directly or indirectly – affected upon drug-binding. By use of mass spectrometry, the thermal profiles of affected proteins can be determined and thus respective drug-targets successfully identified [22, 23].

**Fig. 7:**
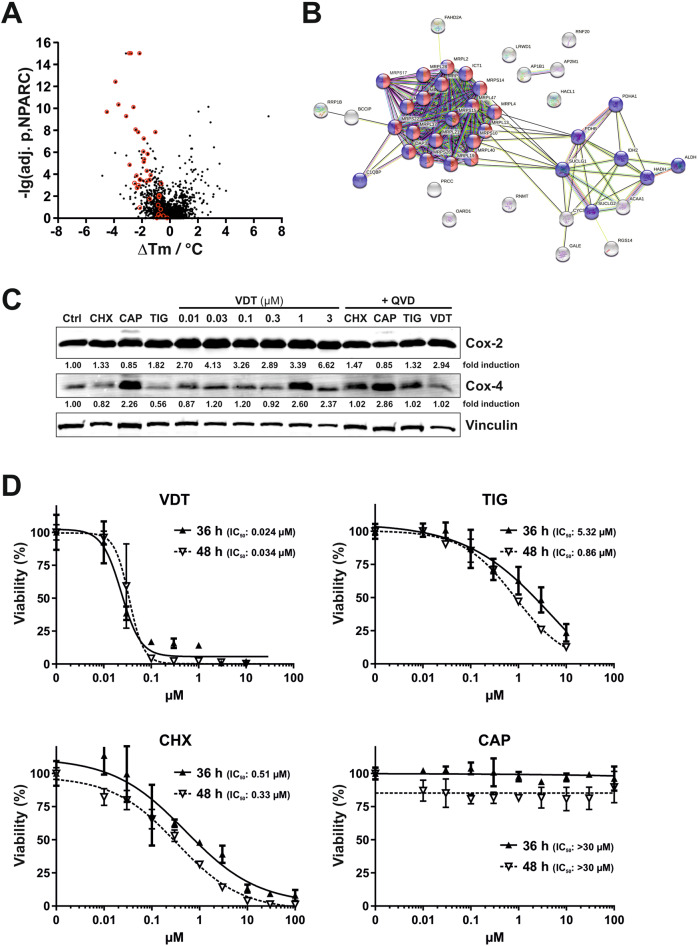
Thermal proteome profiling of potential VDT target proteins and effect of the inhibition of mitochondrial and cytosolic translation on protein expression and viability. **A** For mass spectrometry based thermal proteome profiling (TPP), Ramos cells were treated with 10 µM VDT or diluent control for 30 min. The statistical significance for the VDT-induced difference in protein melting behavior (expressed as the negative decadic logarithm of the adjusted NPARC *p*-value, -lg(adj. p, NPARC)) is plotted against the melting point difference (difference of the means of the melting points, ΔTm). Mitoribosomal proteins are shown in red within the plot. **B** Shown is the functional protein association network (based on the STRING database) of the top 43 proteins destabilized by VDT, selected by *p*-value and melting point difference. Mitochondrial proteins are labeled in blue, mitoribosomal proteins in blue/red and non-mitochondrial proteins in gray. **C** Ramos cells were treated with increasing concentrations of VDT. Cycloheximide (CHX; 1 µM) was used as inhibitor of cytosolic protein translation and chloramphenicol (CAP; 150 µM) and tigecycline (TIG; 10 µM) as inhibitors of mitochondrial translation. To prevent caspase-mediated protein degradation the pan-caspase inhibitor QVD (10 µM) was added before application of cycloheximide (CHX; 1 µM), chloramphenicol (CAP; 150 µM), tigecycline (TIG; 10 µM) or viriditoxin (VDT; 3 µM; right lane). After 36 h immunoblotting against cytochrome c oxidase 2 and 4 (Cox-2 and Cox-4) was performed. Cox-2 is translated within the mitochondria whereas Cox-4 is translated in the cytosol. Immunoblotting for vinculin was used as loading control. Numbers under Cox-2 and Cox-4 immunoblots indicate densitometric analyses of the fold induction of Cox-2/-4 relative to loading control (vinculin). **D** Ramos cells were treated with increasing concentrations of VDT, cycloheximide (CHX), chloramphenicol (CAP), or tigecycline (TIG) for 36 h and 48 h, respectively. Subsequently, cell viability was monitored by AlamarBlue® assay. Mean ± SD values of triplicates are shown. Respective IC_50_ values are given in parenthesis.

Using the TPP-approach we could identify 43 proteins in Ramos cells with a reduced thermal stability upon VDT treatment of whom 29 were of mitochondrial origin (see Table 1 and Fig. 7A, B). Interestingly, several enzymes of the tricarboxylic acid (TCA, Krebs) cycle (such as two subunits of the pyruvate dehydrogenase complex (PDHA1, PDHB), two succinate-CoA ligase subunits (SUCLG1, SUCLG2) and isocitrate dehydrogenase 2 (IDH2)) were affected upon VDT treatment. Since all these enzymes require NAD^+^ and produce NADH plus H^+^ we measured the NAD/NADH ratio. However, using high-performance liquid chromatography and an enzymatic activity assay we observed that VDT did not affect the NAD/NADH ratio whereas – as to be expected – the ratio was reduced upon treatment with the ETC complex I inhibitor rotenone (Supplementary Fig. 4A, B). Thus, we conclude that the respective TCA enzymes are obviously not affected by VDT.

**Table 1:** List of proteins with reduced thermal stability upon VDT treatment.

MRPL (mitochondrial ribosomal proteins; large 39S-subunit)		-lg(adj. p, NPARC)	ΔTm
MRPL2	CGI-22, MRPL14, RPML14	10.32	−3.68
MRPL4	CGI-28	inf.	−2.19
MRPL13	L13, L13A, L13mt, RPL13, RPML13	inf.	−2.80
MRPL15	HSPC145, L15mt, MRP-L15, MRPL7, RPML7	7.82	−2.27
MRPL19	KIAA0104, MRP-L15, RLX1, RPML15	3.87	−2.39
MRPL21		12.41	−3.91
MRPL28	MAAT1, p15	inf.	−2.91
MRPL37	MRPL2, RPML2	9.65	−4.53
MRPL38	HSPC262, MGC4810, MRPL3, RPML3	7.24	−1.87
MRPL40	MRPL22, NLVCF	5.81	−1.60
MRPL47	CGI-204, NCM1	5.34	−1.83
MRPL58	Peptidyl-tRNA hydrolase; ICT1, DS-1	10.10	−2.63
MRPS (mitochondrial ribosomal proteins; small 28S-subunit)			
MRPS10	FLJ10567	7.82	−1.05
MRPS14	HSMRPS14	5.10	−1.89
MRPS15	FLJ11564	4.84	−2.89
MRPS17	HSPC011, RPMS17	4.30	−1.66
MRPS18B	C6orf14, HSPC183, MRPS18-2, PTD017	6.05	−1.92
MRPS22	C3orf5, GIBT, GK002, MRP-S22	9.25	−3.13
MRPS26	C20orf193, dJ534B8.3, MRPS13, MRPS26, RPMS13	8.05	−2.47
MRPS29	DAP3, bMRP10, DKFZp686G12159, MGC126058, MGC126059	3.89	−1.39
Other mitochondrial proteins			
ALDH2	Aldehyde dehydrogenase 2	5.48	−1.11
C1QBP	Complement component 1 Q binding protein; gC1Q-R, gC1qR, HABP1, p32, SF2p32	4.24	−1.08
CYCS	CYC, HCS; cytochrome c	3.86	−4.85
HADH	Hydroxyacyl-CoA dehydrogenase; HADH1, HADHSC, SCHAD	5.95	−1.61
IDH2	Isocitrate dehydrogenase 2	6.10	−2.22
PDHA1	Pyruvate dehydrogenase E1, α-subunit	5.80	−2.05
PDHB	Pyruvate dehydrogenase E1, β-subunit	Inf.	−3.16
SUCLG1	Succinate-CoA ligase, α-subunit	6.33	−1.43
SUCLG2	Succinate-CoA ligase, β-subunit	4.5	−1.03
Other non-mitochondrial proteins			
ACAA1	Acetyl-CoA acyltransferase 1; peroxisomal	4.32	−1.27
AP1B1	Adaptor related protein complex 1, β-subunit; ADTB1, AP105A, BAM22, CLAPB2	4.66	−0.54
AP2M1	Adaptor related protein complex 2 mu 1; AP50, CLAPM1, mu2	4.32	−0.62
BCCIP	BRCA2 and CDKN1A interacting protein; TOK-1	5.39	−1.04
FAHD2A	Fumarylacetoacetate hydrolase domain-containing protein 2A; CGI-105	5.73	−3.14
GALE	UDP-glucose-4-epimerase; SDR1E1	4.37	−1.45
HACL1	2-hydroxyacyl-CoA lyase 1; 2-HPCL, HPCL, PHYH2	5.95	−3.94
LRWD1	Leucine-rich repeats & WD repeat-containing protein 1; CENP-33, ORCA	4.24	−1.33
OARD1	O-acyl-ADP-ribose deacylase 1; C6orf130, dJ34B21.3, MGC19570, TARG1	5.49	−2.03
PRCC	Papillary renal cell carcinoma; RCCP1	6.12	−1.24
RGS14	Regulator of G-protein signaling 14	4.60	−1.96
RNF20	Ring finger protein 20; BRE1, BRE1A, FLJ11189, FLJ20382, hBRE1, KAIA2779	6.82	−1.62
RNMT	RNA guanine-7 methyltransferase; RG7MT1	4.05	−2.10
RRP1B	Ribosomal RNA processing protein 1B; KIAA0179, Nnp1, PPP1R136, RRP1	4.11	−0.76

Ramos cells were incubated with VDT for 30 min and proteins with reduced thermal stability were identified by mass spectrometry based thermal proteome profiling. Proteins with -lg(adj. p, NPARC) > 3.85 and ΔTm <0 were regarded as significantly destabilized. Alternative denominations are shown in the second column.

Intriguingly, 20 candidates were mitochondrial ribosomal (mitoribosomal) proteins belonging to the large 39S-subunit (12 proteins) or small 28S-subunit (8 proteins; see Table 1 and Fig. 7A, B). In this context, targeting mitochondrial translation by tigecycline has been shown to inhibit ETC complexes I and IV and to selectively kill leukemia stem and progenitor cells [6, 24]. Thus, it was conceivable that VDT – like tigecycline – might target the mitochondrial translation and thereby affect the mitochondrial respiratory chain [24].

To investigate whether VDT affects the function of mitoribosomes we monitored the expression of proteins whose translation is dependent on mitochondrial or cytosolic ribosomes. Therefore, we incubated Ramos cells with increasing concentrations of VDT for 36 h and investigated the expression of cytochrome c oxidase 2 and 4 (Cox-2 and Cox-4, respectively). Cox-2 and Cox-4 are subunits of the respiratory complex IV in the mitochondrial electron transport chain. However, in contrast to Cox-2 that is translated by mitochondrial ribosomes, Cox-4 is translated by cytosolic ribosomes [25]. As controls we used cycloheximide for the inhibition of cytosolic translation and chloramphenicol or tigecycline as known inhibitors of mitochondrial protein translation [24]. To exclude a caspase-mediated proteolytic effect on protein expression we additionally applied the pan-caspase inhibitor QVD. As shown in Fig. 7C, VDT and tigecycline did not decrease the expression of mitochondrial translated Cox-2 after 36 h – though in Fig. 6D, E we observed a minor reduction of Cox-2 upon 24 h of VDT treatment. Likewise, chloramphenicol only slightly reduced the expression of Cox-2 (Fig. 7C). All in all, we observed a rather stable protein expression of Cox-2 in Ramos cells after 36 h that was not substantially affected by VDT or tigecycline (Fig. 7C). Considering the stable expression of mitochondrial translated Cox-2, we rather excluded an effect of VDT on mitochondrial protein translation as the primary mechanism for apoptosis induction, since VDT induced the activation of caspases already after 3–4 h (Fig. 2A, B). This could be further corroborated by the observation that inhibition of mitochondrial translation by chloramphenicol (in contrast to inhibition of cytosolic translation by cycloheximide) did not display any cytotoxic effect in Ramos cells – even after 48 h of incubation (Fig. 7D). VDT however, induced cytotoxicity at lower concentrations and much more pronounced as tigecycline.

## Discussion

The mycotoxin VDT is a natural product that so far has been known primarily for its antibiotic effects and whose mechanisms of action in eukaryotic cells are not yet fully understood. In this study, we could identify VDT as a highly potent and rapid apoptosis inducer in human lymphoma and leukemia cells. We show that this effect is based on the multifaceted intervention of VDT in mitochondrial functionality, which leads to a collapse of mitochondrial respiration (most likely by inhibition of ETC complex I), the breakdown of the mitochondrial membrane potential (ΔΨm), mitochondrial cytochrome c release, ROS production and fragmentation of the mitochondrial network. Most interestingly, our studies show that VDT’s specific activity profile opens a therapeutic window for the treatment of hematological neoplasia, making it a promising lead structure for the development of novel anticancer drugs. Finally, using a thermal proteome profiling (TPP) approach, we could demonstrate that mitochondrial ribosomes are affected (destabilized) by VDT.

A prominent feature of VDT is its capacity to activate the mitochondrial apoptosis pathway in the presence of Bcl-2 or the absence of Bax and Bak. Antiapoptotic members of the Bcl-2 family such as Bcl-2 (B cell leukemia or lymphoma gene number 2), Bcl-xL (B cell lymphoma-extra large), or Mcl-1 (myeloid cell leukemia sequence 1) have first been identified in leukemia and lymphoma and are also frequently overexpressed in other types of neoplasia to inhibit apoptotic cell death during tumorigenesis. Moreover, since the major mechanism of genotoxic radio- and chemotherapy is the p53-mediated activation of the mitochondrial apoptosis pathway, tumor cells gain therapy resistance by inactivating this pathway via overexpression of anti-apoptotic Bcl-2 proteins [15, 26]. We could show that VDT induced apoptosis in the presence of Bcl-2 and was blocked in Jurkat cells deficient for caspase-9 (as the central initiator caspase of the mitochondrial apoptosis pathway; see Fig. 4). However, VDT – unlike staurosporine [14] – did not activate caspase-9 independent of apoptosome formation since caspase-9 activation was blocked in Jurkat cells expressing a mutant form of caspase-9 (R56A) [27] that disables Apaf-1 binding (data not shown). Thus, VDT is capable of directly activating the mitochondrial apoptosis pathway even in the presence of Bcl-2 and therefore represents a valuable agent for the treatment of therapy-resistant neoplasia.

Another intriguing feature of VDT is its specificity for leukemia and lymphoma. VDT was highly cytotoxic in human Ramos lymphoma cells and in the human leukemia cell lines Jurkat HL60, HPBALL, K562, KOPTK1, MOLT4, and SUPB15 (Fig. 1F, Supplementary Fig. 1) as well as in the murine lymphoma cell line L5187Y [9], whereas all tested solid tumors cell lines (143B, HCT116, HeLa, HT29. MCF7, RT112, SH-SY5Y) were far less susceptible (Fig. 1E, F). This goes in line with previous reports that tested VDT in solid tumor cell lines (A549, DU145, HCT116, KB, LNCaP, PC3, SH-SY5Y, SK-OV3) and also observed only a moderate cytotoxicity [8, 12, 13]. Likewise, human hematopoietic stem and progenitor cells (HSPC) and peripheral blood mononuclear cells (PBMC) were rather resilient to VDT treatment. An evident explanation for this phenomenon might be that mitochondrial mass and metabolism are increased in leukemia and lymphoma compared to normal HSPC [24, 28–34]. In addition, an increased mitochondrial metabolism renders leukemia resistant to conventional genotoxic therapies as well as targeted therapies [35, 36]. Therefore, among the different cancer entities, leukemia and lymphoma seem to be especially vulnerable to targeting of the mitochondrial metabolism [4, 6, 37, 38].

A potent driver of mitochondrial biogenesis is the oncogene *c-myc* and consequently increased c-Myc expression represents a hallmark of Burkitt lymphoma and B cell and T cell acute lymphoblastic leukemias (ALL) [31] which might explain the high sensitivity of the Burkitt lymphoma cell line Ramos to VDT-induced cytotoxicity. In this context, Skrtic et al. could show that targeting mitochondrial translation by tigecycline inhibits ETC complexes I and IV and selectively targets leukemia stem and progenitor cells due to their increased mitochondrial mass [6, 24]. In addition, by *c-myc* repression, they could show that only in cells with functional c-Myc and thus increased mitochondrial mass, tigecycline displayed its cytotoxic potential [24].

In contrast to VDT, conventional drugs like paclitaxel or vinblastine affected the colony-forming potential of HSPC at very low concentrations (Fig. 3C) that corresponds to their hematotoxic side effects. Therefore, VDT is apparently superior to these common cytostatic drugs with regard to their hematotoxicity profile.

Furthermore, we could show that the mitochondrion is the prime target of VDT as monitored by OXPHOS inhibition, the decrease of ΔΨm and ATP level, mitochondrial cytochrome c release, and ROS generation which coincided with the loss of L-OPA1 and subsequent mitochondrial fission. Maintaining the mitochondrial membrane potential (ΔΨm) is crucial for the cell, as it drives essential processes such as ATP synthesis [39] and depolarization below a certain threshold is accordingly associated with a loss of cell viability [40]. Similar effects on ΔΨm have been described for a variety of substances like the protonophore CCCP [41] or ETC inhibitors [42, 43]. However, while CCCP in high concentrations causes an abrupt collapse of ΔΨm by shuttling protons through the inner mitochondrial membrane, inhibitors of ETC complexes I - IV mediate a slow decrease of ΔΨm [44] as observed for VDT (Fig. 4F).

VDT caused a rapid and substantial inhibition of the mitochondrial respiration, suggesting that the demonstrated decrease in ΔΨm and ATP levels was due to an inhibition of OXPHOS. Accordingly, we could show that VDT reduced the activity of complex I of the respiratory chain. Consequently, VDT-mediated inhibition of ETC complex I and concomitant decrease in ATP level might be responsible for cell death induction since Izyumov et al. could show that even a transient three-hour drop of the ATP level to 30% induces apoptosis [45].

It has been shown that the rapid breakdown of ΔΨm and decrease of cellular ATP level can cause the proteolytic processing of OPA1 by OMA1 leading to loss of fusion-competent L-OPA1 [46–48]. This finally shifts the balance towards mitochondrial fission [49–51] and can ultimately result in cell death [19]. Mitochondrial fission is an early event during apoptosis, occurring before caspase activation and membrane blebbing and coincides with the mitochondrial release of cytochrome c [52]. Accordingly, upon treatment with VDT, the loss of L-OPA1 coincided with the impairment of ΔΨm, ATP levels and subsequent apoptosis induction.

Adverse alterations in mitochondrial morphology and functionality are frequently associated with an increased ROS level, which is primarily described as a consequence of partial inhibition of the ETC [53–55] or mitochondrial fragmentation [56]. We could show that VDT caused an increase in cellular ROS levels and that co-treatment with the antioxidant NAC rescued the levels of L-OPA1, resulting in decreased cytotoxicity. Therefore, we conclude that the axis of VDT-induced mitochondrial damage, fragmentation and apoptotic cell death is at least partially ROS-dependent.

In order to identify proteins affected by VDT, we performed a thermal proteome profiling (TPP) and could identify 29 proteins of mitochondrial origin that were destabilized by VDT, of whom 20 were part of the mitochondrial ribosome (see Table 1). Thus, it was conceivable that VDT might display similar features as tigecycline that selectively kills leukemia stem and progenitor cells by targeting mitochondrial translation [24]. Though, mitochondria contain more than 1100 proteins, only 13 of the 90 proteins of the mitochondrial respiratory chain are encoded by mitochondrial DNA and translated by mitochondrial ribosomes (mitoribosomes) within the mitochondrial matrix [57]. Therefore, inhibition of mitochondrial metabolism via targeting mitochondrial translation represents a promising therapeutic approach [4, 6, 24, 38].

However, VDT did not show a pronounced effect on mitochondrial encoded protein synthesis. This might be attributed to a stable expression and slow turnover rate of mitochondrial translated proteins in Ramos cells since the mitochondrial protein synthesis inhibitors chloramphenicol and tigecyclin displayed also no prominent effect on the expression of mitochondrial translated Cox-2 after 36 h. Accordingly, due to the rapid kinetics of VDT-induced apoptosis (within 3–4 h; Fig. 2A, B) we conclude that the apoptotic mechanism is rather not attributed to inhibition of the mitochondrial translation of ETC proteins. In addition, inhibition of mitochondrial translation via chloramphenicol did not affect the viability of Ramos cells even after 48 h treatment (Fig. 7D).

The detection of 20 destabilized proteins within the mitoribosome via TPP approach does not necessarily mean that all these proteins are directly targeted by VDT. Instead, it is rather conceivable that VDT affects the overall stability of mitoribosomes by targeting single protein(s) upstream or within the ribosome that are crucial for its stability or association with the inner mitochondrial membrane. In addition to their function in mitochondrial protein synthesis, mitoribosomes are also associated with mitochondrial dynamics, morphology and intrinsic apoptosis. For example, the loss of the mitoribosomal protein DAP3 (death-associated protein 3, MRPS29) leads to a collapse of ΔΨm, a decrease in ATP-level, fragmentation of mitochondria and a sensitization to intrinsic apoptosis [58]. Interestingly, DAP3 is also destabilized by VDT (see Table 1). Thus, VDT might act in a similar way as actinonin that has been shown to disrupt the mitoribosome intactness and to cause mitochondrial fragmentation [59].

Mitoribosomes have been reported to associate with the inner mitochondrial membrane via prohibitins [60] and depletion of prohibitins, in turn, leads to OPA1 processing and subsequent mitochondrial fragmentation [61], indicating that the interaction of mitoribosomes with the inner membrane plays a crucial role in maintaining mitochondrial stability. As part of the mitoribosome, DAP3 can interact with the inner membrane protein hNOA1 that is a binding partner of ETC complex I [62]. Knockdown of hNOA1 reduces mitochondrial O_2_ consumption in an ETC complex I-dependent manner, supporting a functional link between hNOA1 and complex I [62]. Therefore, it has been suggested that DAP3 might exert a similar function as that of prohibitins to bridge mitoribosomes and inner membranes [58]. In this context, depletion of DAP3 might exert the same effect as VDT-mediated targeting of DAP3. It is therefore conceivable that the VDT-mediated destabilization of mitoribosomes and subsequent detachment from prohibitins or hNOA1 leads to the loss of long isoforms of OPA1 and subsequent mitochondrial fission accompanied by a collapse of ΔΨm, inhibition of ETC complex I, decrease in ATP-level, and ultimately to the induction of apoptosis.

Taken together, we could demonstrate that the mitotoxin VDT joins a new generation of anticancer drugs that target the mitochondrial metabolism for the treatment of leukemia and lymphoma. VDT potently induced apoptosis in nanomolar range and in rapid kinetics in leukemia and lymphoma cells by causing inhibition of OXPHOS, breakdown of ΔΨm, mitochondrial cytochrome c release, oxidative stress and fragmentation of the mitochondrial network. The peculiar feature of VDT is (i) its high specificity for lymphoma and leukemia cells, (ii) its low cytotoxicity in untransformed HSPC and PBMC which offers a therapeutic window, and (iii) its proficiency to directly activate the mitochondrial apoptosis pathway even in the presence of Bcl-2 which enables overcoming of therapy resistance in hematological neoplasia that express antiapoptotic Bcl-2 proteins.

## Materials and methods

### Extraction and isolation of VDT

VDT was isolated as described before [9]. Briefly, the fungus *Cladosporium cladosporioides* was isolated from the sediment of a hypersaline lake in Egypt and cultivated on solid rice medium. After 35 d the fungal culture was extracted with EtOAc. Subsequently, VDT was purified from the crude extract via liquid-liquid partitioning, vacuum liquid chromatography, column chromatography and reversed-phase HPLC. In order to exclude any unwanted metabolization or isomerization we prepared VDT in small, lyophilized aliquots and dissolved it in DMSO immediately before use.

### Reagents

Antimycin A (#A8674), carbonyl cyanide m-chlorophenyl hydrazone (CCCP, #C2759), chloramphenicol (#C0378-5G), clotrimazol (#C6019), lonidamine (#L4900), paclitaxel (#T7402), potassium cyanide (#60178), rotenone (#45656), sodium azide (NaN_3,_ #S200), thenoyltrifluoroacetone (TTFA, #88300), tigecycline (#220620097) and vinblastine (VBL, #V1377) were purchased from Sigma (Munich, Germany), cycloheximide (#8682.1) from Roth (Karlsruhe, Germany), N-(2-Quinolyl)valyl-aspartyl-(2,6-difluorophenoxy)methyl ketone (QVD, #S7311) from Selleckchem (Houston, TX, USA), oligomycin A (#O532970) from Toronto Research Chemicals (Toronto, Canada), etoposide (#1043) from Biovision and staurosporine (STS, #9300) from LC Laboratories (Woburn, MA, USA). All other substances for which a manufacturer is not explicitly specified were obtained from Carl Roth.

### Cell lines, primary cells, and cell culture

DG75 cells (human B cell Burkitt lymphoma; #ACC-83), HeLa cells (human cervix carcinoma; #ACC-57), HT29 cells (human colon carcinoma; #ACC-299), HCT116 cells (human colon carcinoma; #ACC-581), Jurkat (JM) cells (human T cell acute lymphoblastic leukemia; #ACC-282), MCF7 cells (human breast carcinoma; #ACC-115), RT112 cells (human urinary bladder carcinoma #ACC-418), SH-SY5Y cells (human neuroblastoma; #ACC-209), HL60 (human acute myeloid leukemia; #ACC-3), HPBALL (human T cell acute lymphoblastic leukemia; #ACC-483), MOLT4 (human T cell acute lymphoblastic leukemia; #ACC-362), K562 (human chronic myeloid leukemia; #ACC-10), and SUPB15 (human B cell acute lymphoblastic leukemia; #ACC-389) were obtained from DSMZ and 143B cells (human osteosarcoma; #CRL-8303) from ATCC. KOPTK1 cells (human T cell acute lymphoblastic leukemia; #CVCL_4965) were kindly provided by Oskar Haas (Children’s Cancer Research Institute, St. Anna Children’s Hospital, Vienna, Austria). Ramos cells (human B cell Burkitt lymphoma) were kindly provided by Michael Engelke (Institute of Cellular and Molecular Immunology, University Hospital Göttingen, Göttingen, Germany). HeLa cells stably expressing mito-DsRed were kindly provided by Aviva M. Tolkovsky (Department of Clinical Neurosciences, University of Cambridge, England, UK) and have been described previously [63]. Caspase-9 deficient Jurkat (JMR) cells were kindly provided by Klaus Schulze-Osthoff (Interfaculty Institute for Biochemistry, University of Tübingen, Tübingen, Germany) retrovirally transduced with either empty pMSCVpuro (Clontech, Heidelberg, Germany) or pMSCVpuro containing cDNAs coding for untagged human wild-type caspase-9 as previously described [14]. Jurkat (E6) cells expressing wild-type Bcl-2 and respective vector control cells were kindly provided by Claus Belka (Ludwig-Maximilians University, Munich, Germany). Caspase-8 deficient Jurkat cells and the parental cell line A3 were kindly provided by John Blenis (Sandra and Edward Meyer Cancer Center, New York, NY, USA). Jurkat, Ramos, HL60, HPBALL, MOLT4, K562, SUPB15, and KOPTK1 cells were cultivated in RPMI 1640 medium supplemented with 10% FCS, 100 U/ml penicillin, and 100 µg/ml streptomycin at 37 °C and 5% CO_2_ in a humidity-saturated atmosphere. HCT116, HT29, MCF7, 143B, and RT112 cells were cultivated under the same conditions but in Dulbecco’s Modified Eagle’s medium (DMEM) high-glucose instead of RMPI 1640. Peripheral blood mononuclear cells (PBMC) were obtained from the blood of healthy donors by apheresis and subsequent density gradient centrifugation. Hematopoietic stem and progenitor cells (HSPC) were separated by immunomagnetic cell separation (MACS, Miltenyi Biotec, Bergisch–Gladbach, Germany) using an antibody against the stem cell marker CD34. HSPC cells were subsequently cultivated under the same conditions as described for Ramos and Jurkat cells, but with an additional supplementation of cytokines [Interleukin 3 (IL-3), interleukin 6 (IL-6), stem cell factor (SCF), Flt3-ligand, 10 ng/ml each, all purchased from PreproTech GmbH, Hamburg, Germany]. The study of PBMC and HSPC was approved by the local ethical review committee (study number: 5944 R; registration ID: 2017044215), and all patients gave written informed consent.

### Cytotoxicity measurements

For the measurement of cytotoxicity in Ramos and Jurkat cells, resazurin reduction assay (also known as AlamarBlue® assay) was performed. In short, cells were seeded at a certain density depending on the intended incubation time (5 × 10^5^ cells/ml for 8 or 24 h, 1 × 10^5^ cells/ml for 72 h), treated with ascending substance concentrations and after the specified incubation time, resazurin (Sigma, #R7017) was added to a final concentration of 40 µM. After 90 min of incubation, the fluorescence of resorufin (excitation: 535 nm, emission: 590 nm) was measured with a microplate spectrophotometer. Since the reduction of resazurin to resorufin is proportional to aerobic respiration it serves as a measure of cell viability. For technical reasons, the MTT assay was used for the determination of cytotoxicity in solid tumor cell lines and HSPC, after an initial evaluation that proved both assays provided comparative results concerning the cytotoxicity of VDT. In principle, the procedure is similar to that already described for the AlamarBlue® assay. However, the cell densities were adjusted (1 × 10^5^ cells/ml for solid cancer cell lines, 4 × 10^5^ cells/ml for HSPC) and the cells were loaded with 1 mg/ml 3-(4,5-dimethylthiazol-2-yl)-2,5-diphenyltetrazolium bromide (MTT, Sigma, #M2128) instead of resazurin for 60 min after treatment with the test substance. Subsequently, the formazan crystals formed were solubilized with DMSO and absorbance was measured (test wavelength: 570 nm, reference wavelength: 650 nm). For both assays, the viability of cells treated with DMSO (0.1% v/v) was set to 100% and the dose-response curves were then fitted with Prism v7.01 (GraphPad Software, La Jolla, CA, USA).

### Fluorometric caspase-3 activity assay

Ramos or Jurkat cells were seeded at a density of 1 × 10^6^ cells/ml and treated with the respective agents for the indicated time. Briefly, cells were harvested by centrifugation at 600 g and lysed with 50 μl of ice-cold lysis buffer (20 mM HEPES, 84 mM KCl, 10 mM, MgCl_2_, 200 μM EDTA, 200 μM EGTA, 0.5% NP40, 1 μg/ml leupeptin, 1 μg/ml pepstatin, 5 μg/ml aprotinin) on ice for 10 min. Cell lysates were transferred to a black flat-bottom microplate and mixed with 150 μl of ice-cold reaction buffer (50 mM HEPES, 100 mM NaCl, 10% sucrose, 0.1% CHAPS, 2 mM CaCl_2_, 13.35 mM DTT) containing 70 μM of the profluorescent caspase substrate Ac-DEVD-AMC (Biomol GmbH, Hamburg, Germany, #ABD-13402). The kinetics of AMC release was monitored by measuring AMC fluorescence intensity (excitation: 360 nm, emission: 450 nm) at 37 °C in intervals of 2 min over a time course of 150 min, using a Synergy Mx microplate reader. The slope of the linear range of the fluorescence curves (Δrfu/min) was considered as corresponding to caspase-3 activity. To correct for differences in endogenous caspase activity between independent experiments, all values were normalized in a two-step process. In the first step, all values were divided by the mean of all values measured at the respective time point. In the second step, this value was then divided by the mean of the solvent controls of all independent experiments at the respective time point.

### FACS-based analysis of apoptotic cell death and cell cycle

The leakage of fragmented DNA from apoptotic nuclei and cell cycle analysis was measured by the method of Nicoletti et al. [64]. Briefly, nuclei were prepared by lysing cells in a hypotonic lysis buffer (1% sodium citrate, 0.1% Triton X-100, 50 µg/ml propidium iodide) and subsequently analyzed by flow cytometry. Nuclei to the left of the 2 N peak containing hypodiploid DNA were considered as apoptotic. All flow cytometric analyses were performed on an LSR-Fortessa™ (Becton Dickinson, Heidelberg, Germany).

### Immunoblotting

Ramos or Jurkat cells were treated as specified and then harvested by centrifugation (1000 g, 5 min) and quick-frozen in liquid nitrogen. Cell pellets were thawed on ice, incubated in lysis buffer [20 mM Tris-HCl, 150 mM NaCl, 1% v/v Triton X-100, 0.5 mM EDTA, 1 mM Na_3_VO_4_, 10 mM NaF, 2.5 mM Na_4_P_2_O_7_, 0.5% sodium deoxycholate, protease inhibitors (Sigma, #P2714)] for 30 min. Cell lysates were purified from cell debris by centrifugation (20,000 g, 15 min) and the protein concentration in the supernatant was determined by Bradford assay. The samples were diluted to a homogeneous protein concentration with sample buffer and both SDS-PAGE and Western blot analyses were performed. Finally, target protein specific primary antibodies [anti-OPA1, anti-PARP1 (Enzo, #BML-SA250), anti-Caspase-8 (Cell Signaling Technology, #9746), anti-caspase-9 [14], anti-tubulin (TUBA4A; Sigma, #T5168), anti-vinculin (Sigma, #V9131), anti-Cox-4 (cytochrome c oxidase-4; Proteintech, #55070-1-AP), or total OXPHOS Human WB Antibody Cocktail (Abcam, #ab110411; consisting of anti-NDUFB8 (NADH ubiquinone oxidoreductase subunit B8), anti-SDHB (succinate dehydrogenase subunit B), anti-UQCRC2 (ubiquinol-cytochrome c reductase core protein 2), anti-Cox-2 (cytochrome c oxidase-2), anti-ATP5A (ATP synthase F1α), and anti-cytochrome c (BD Bioscience, BD556433)] were applied and fluorescence-coupled secondary antibodies (LI-COR Biosciences) were used for protein detection on a PVDF membrane using the LI-COR Odyssey® imaging system.

### Mitochondrial cytochrome c release

5 × 10^6^ Ramos cells were treated with 0.1% DMSO (0.1% v/v), 1 µM VDT, or 2.5 µM STS for 8 h. Cells were harvested by centrifugation (1200 rpm, 5 min). The pellets were incubated in lysis buffer (0.025% digitonin, 20 mM HEPES pH 7.5, 100 mM sucrose, 2.5 mM MgCl_2_, 100 mM KCl; supplemented with protease inhibitors) on ice for 10 min followed by centrifugation at 14,000 g for 10 min at 4 °C. The supernatant containing the cytosolic fraction was transferred to a new reaction tube and an appropriate amount of sample buffer was added. Samples were then analyzed by SDS-PAGE and immunoblot as described above.

### Densiometric analyses

Immunoblots were quantified using the LI-COR software Image Studio Lite Ver 5.2. The signals of every densiometric band of the proteins of interest and the loading control were determined. The fold induction represents the signal of the protein of interest, which was normalized to the corresponding loading control and set in relation to the level of the protein of interest from the control (DMSO). The detection and determination of ratios, such as PARP uncleaved p116 to PARP cleaved p85, as well as OPA1 long forms 1,2 (OPA1L1,2) to OPA1 short forms 3-5 (OPA1S3-5) were determined as follows. The signals of every densiometric band of the proteins of interest (p116 and p85) or (OPA1L1,2 and OPA1S3-5) and the loading control were determined. p116 was normalized to its corresponding loading control and likewise for p85 or (OPA1L1,2 and OPA1S3-5). The shown values represent the calculated ratio from p116/(p116 + p85) and OPA1L1,2/(OPA1L1,2 + OPAS3-5), respectively.

### Microscopy and quantification of mitochondrial fragmentation (fission)

Imaging of HeLa cells stably expressing mito-DsRed was performed using a spinning disc confocal microscope (Perkin-Elmer, Waltham, MA, USA) equipped with a 60x/1.4 NA oil-immersion objective using a 488 nm laser line for excitation. The cells were seeded on glass bottom 3 cm dishes (MatTek, Ashland, MA, USA) and maintained in full growth medium supplemented with 10 mM HEPES during imaging. Images were taken for a duration of 30 to 45 min in a chamber heated to 37 °C. Image analysis was performed using Fiji. 3D stacks were compressed with max projection and brightness was adjusted for optimal visibility of mitochondria. At least 20 cells per condition were analyzed in detail. At least 30 mitochondria per cell were manually measured for their major and minor axis. For branched mitochondria, only the longest path was measured. Mitochondria with a major axis of less than 0.5 µm were ignored since those signals could originate from mitochondria derived vesicles. Mitochondrial aspect ratio was determined by dividing minor axis by major axis for each mitochondrion and the arithmetic mean for each cell was calculated. Additionally, all imaged cells were categorized in to three categories (tubular, intermediate, and fragmented) after blinding by renaming files with random numbers.

### Colony-forming unit (CFU) assay of healthy hematopoietic stem and progenitor cells (HSPC)

HSPC were derived from healthy donors as described above. The cells were then seeded in 12-well plates with a density of 400 cells/ml and a final volume of 400 µl per well in semisolid ready-to-use methylcellulose growth medium (MethoCult™ SF H4436; Stem Cell Technologies, Vancouver, BC, Canada). After incubation for 14 days at 37 °C and 5% CO_2_ under humidified conditions the resulting colonies were counted and differentiated into colony types via light microscopy (colony types: colony-forming unit-erythroid (CFU-E); burst-forming unit-erythroid (BFU-E); colony-forming unit-granulocyte; colony-forming unit-granulocyte/macrophage; colony-forming unit-macrophage (CFU-G/GM/M), and colony-forming unit-granulocyte/erythrocyte/macrophage/megakaryocyte (CFU-GEMM).

### Measurement of mitochondrial membrane potential

To measure changes in mitochondrial membrane potential (ΔΨm), Ramos cells were loaded with the cell-permeable and positively charged dye tetramethylrhodamine ethyl ester (TMRE), which accumulates in active mitochondria characterized by a negative net charge, whereas depolarized mitochondria do not retain the dye. For this purpose, Ramos cells were resuspended in fresh medium containing 10 mM HEPES and supplemented with 100 nM TMRE (AAT Bioquest, Sunnyvale, CA, USA; #22220). After incubation for 15 min at 37 °C, cells were washed twice with RPMI medium (plus HEPES) and incubated for another 15 min in order to allow the cells to recover and to ensure that the dye had accumulated in active mitochondria. Subsequently, the fluorescence of TMRE was measured by flow cytometry (excitation: 488 nm, emission: 575). First, the basic fluorescence level of active mitochondria was recorded for at least 1 min and then the test substance was added, the sample tube was mixed and the measurement continued for the indicated time. The basic fluorescence level before addition of the test substance was set to 100%. Treatment with the protonophore CCCP served as positive control for complete mitochondrial depolarization.

### Measurement of cellular ROS level

In order to quantify the cellular reactive oxygen species (ROS) level, Ramos cells were cultivated in RMPI medium lacking phenol red for the duration of the experiment. First the cells were loaded with 20 µM 2′,7′-dichlorodihydrofluorescein diacetate (H_2_DCF-DA, Sigma, #D6883) and incubated for 30 min in the dark at 37 °C. Subsequently, cells were washed, seeded in 96-well plates and treated with the test compounds for 6 h. ROS rapidly oxidize H_2_DCF-DA to highly fluorescent 2’,7’-dichlorodichlorodihydrofluorescein (DCF). The fluorescence of DCF, which directly correlates to cellular ROS level, was measured by spectrophotometer (excitation: 485 nm, emission: 530 nm). ROS level of cells treated with DMSO (0.1% v/v) was set to 100%.

### Fluorometric O_2_ consumption assay

Measurement of cellular oxygen consumption rate was performed using the MITO-ID® Extracellular O_2_ Sensor Kit (High Sensitivity) (Enzo Life Sciences, Lörrach, Germany; #51045) according to manufacturer’s instructions. Fluorescence was analyzed using a Synergy Mx microplate reader (excitation: 340–400 nm, emission: 630–680 nm).

### Measurement of cellular ATP levels

Measurements of cellular ATP levels were performed using the mitochondrial ToxGlo™ assay (Promega, Mannheim, Germany; #G8000) according to manufacturer’s instructions. To increase the sensitivity for the detection of mitotoxins, cells were cultivated during measurement in medium containing either glucose or galactose as the only sugar source. Since glycolytic processing of galactose does not produce a net ATP gain, the cells are completely dependent on OXPHOS for ATP production when cultivated in galactose medium.

### Activity measurements for electron transport chain complexes

These measurements were performed using the MitoCheck® complex I-IV activity assay kit (Cayman Chemical, Ann Arbor, MI, USA; #700930/700940/700950/700990) according to manufacturer’s instructions. For each complex, appropriate positive controls were used and the activity of DMSO-treated (0.1% v/v) mitochondria was set to 100%.

### Thermal proteome profiling (TPP)

TPP except MS analysis and data analysis for protein identification and quantification was performed according to Franken et al. [22]. A detailed description is provided in Supplemental Information.

### Replicates and statistical analyses

Experiments were replicated at least three times, and representative data are shown. Error bars indicate standard deviation. All statistical analyses were performed using Prism v7.01 (GraphPad Software, La Jolla, CA, USA).

## Supplementary information

Supplemental Information

Original Data File

Author confirmation concerning changes of author list (see your E-mail from October 5th 2022)

## Data Availability

Data were generated by the authors and included in the article.
