# Supplementary material for: The mycotoxin viriditoxin induces leukemia- and lymphoma-specific apoptosis by targeting mitochondrial metabolism
Source: Cell Death Dis. 2022 Nov 8;13(11):938. doi: 10.1038/s41419-022-05356-w (PMC9643474; doi:10.1038/s41419-022-05356-w)
Supplement: Supplementary file 1 — Supplemental Information [file 41419_2022_5356_MOESM1_ESM.pdf]

Supplemental Information

Supplemental Figures

Supplemental Figure 1: VDT induces cell death in different leukemia cell lines

Supplemental Figure 2: VDT exerts no effect on tubulin polymerization at cytotoxic concentrations

Supplemental Figure 3: VDT does not induce apoptosis via the death receptor pathway

Supplemental Figure 4: VDT does not alter the NAD/NADH ratio

Supplemental Materials and Methods

Thermal proteome profiling (TPP)

Measurement of tubulin polymerization velocity (see Suppl. Fig. 1A, B)

Detection of in situ tubulin polymerization via immunoblotting (see Suppl. Fig. 1C)

Detection of NAD/NADH ratio by enzymatic assay (see Suppl. Fig. 4A)

Detection of NAD/NADH ratio by high-performance liquid chromatography (see Suppl. Fig. 4B)

Supplemental References

Supplemental Figures

Suppl. Figure 1

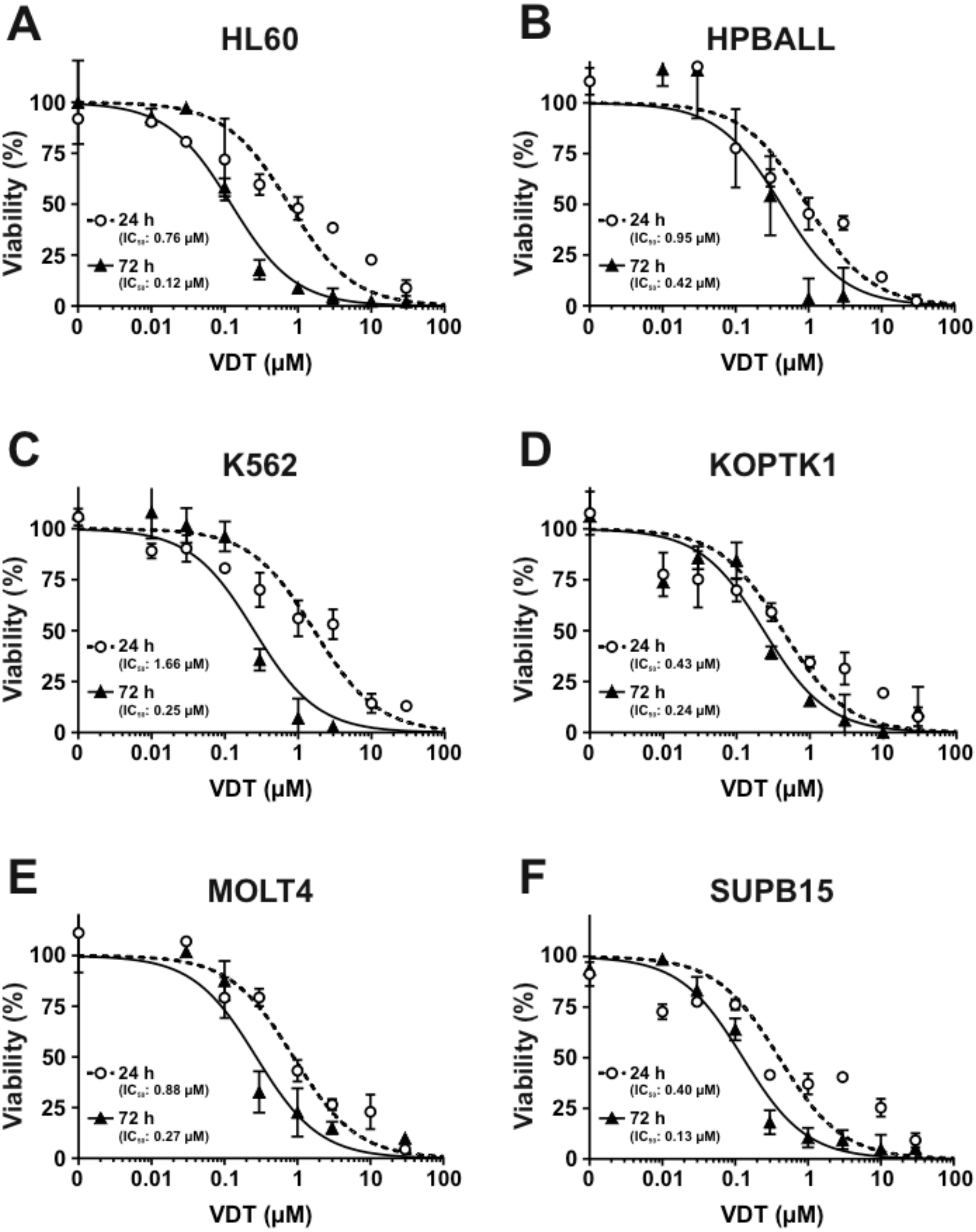

Suppl. Figure 1: VDT induces cell death in different leukemia cell lines

Cytotoxicity was determined after 24 h or 72 h treatment with VDT in (A) HL60 (human acute myeloid leukemia; AML), (B) HPBALL (human T cell acute lymphoblastic leukemia; T-ALL),

(C) K562 (human chronic myeloid leukemia; CML), (D) KOPTK1 (human T-ALL), (E) MOLT4 (human T-ALL), and (F) SUPB15 (human B cell acute lymphoblastic leukemia; B-ALL). Cell viability was assessed by AlamarBlue® viability assay. Mean ± SD values of triplicates are shown. Respective IC_{50} values are given in parenthesis.

Suppl. Figure 2

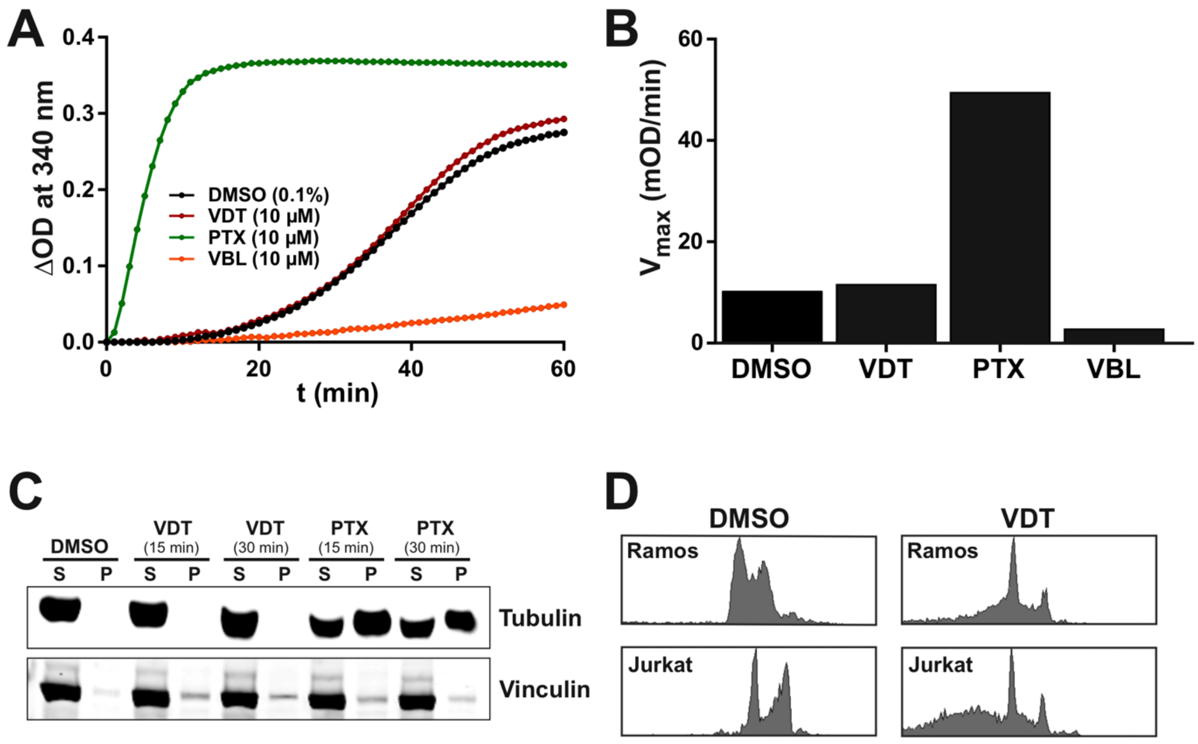

Suppl. Figure 2: VDT exerts no effect on tubulin polymerization at cytotoxic concentrations

(A) Measurement of the polymerization rate of porcine neuronal tubulin under treatment with either 0.1% (v/v) DMSO, 10 µM vinblastine (VBL, as positive control for tubulin destabilization), 10 µM paclitaxel (PXL, as positive control for tubulin stabilization) or 10 µM VDT. Polymerization was started by incubation at 37 °C and followed by absorption monitoring at 340 nm. Absorption at 340 nm is proportional to the concentration of microtubule polymer. (B) Maximum polymerization rates of tubulin calculated based on the data shown in panel A. (C) Soluble (S) and polymerized (P) tubulin from Ramos cells treated with 10 µM VDT or 10 µM PTX was separated by centrifugation and detected via immunoblotting. Immunoblot against vinculin served as control. (D) Ramos or Jurkat cells were treated with 1 µM VDT for 24 h. Subsequently, nuclei were prepared and stained with propidium iodide [1] and the cell cycle distribution was determined via flow cytometry.

Suppl. Figure 3

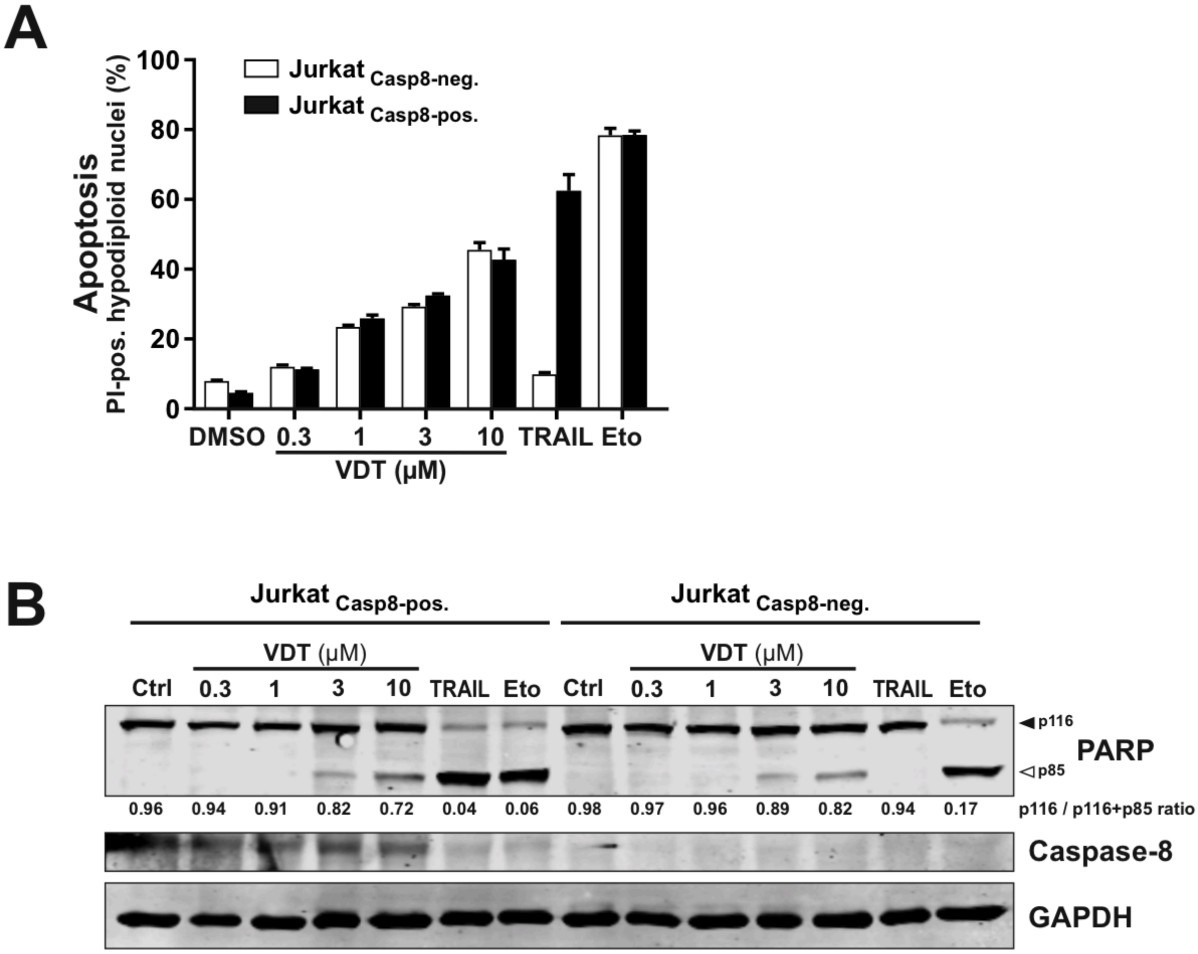

Suppl. Figure 3: VDT does not induce apoptosis via the death receptor pathway

5 x 10^5 (A) or 1 x 10^6 (B) caspase-8 proficient Jurkat cells (Jurkat-Casp8-pos.) or caspase-8 deficient Jurkat cells (Jurkat-Casp8-neg.) were treated with the indicated concentrations of VDT or the death receptor ligand TRAIL (40 ng/ml; positive control). (A) After 24 h, apoptosis was assessed by propidium iodide staining of apoptotic hypodiploid nuclei and flow cytometry. (B) After 8 h, the proteolytic processing of PARP and caspase-8 was detected by immunoblotting. Solid arrowheads indicate the uncleaved form of PARP (p116); open arrowheads indicate the cleaved form (p85). Immunoblotting for glyceraldehyde 3-phosphate dehydrogenase (GAPDH) was used as loading control. Numbers under PARP immunoblot indicate densitometric analyses of the ratio of unfragmented (p116) to total PARP (p116 / p116+p85).

Suppl. Figure 4

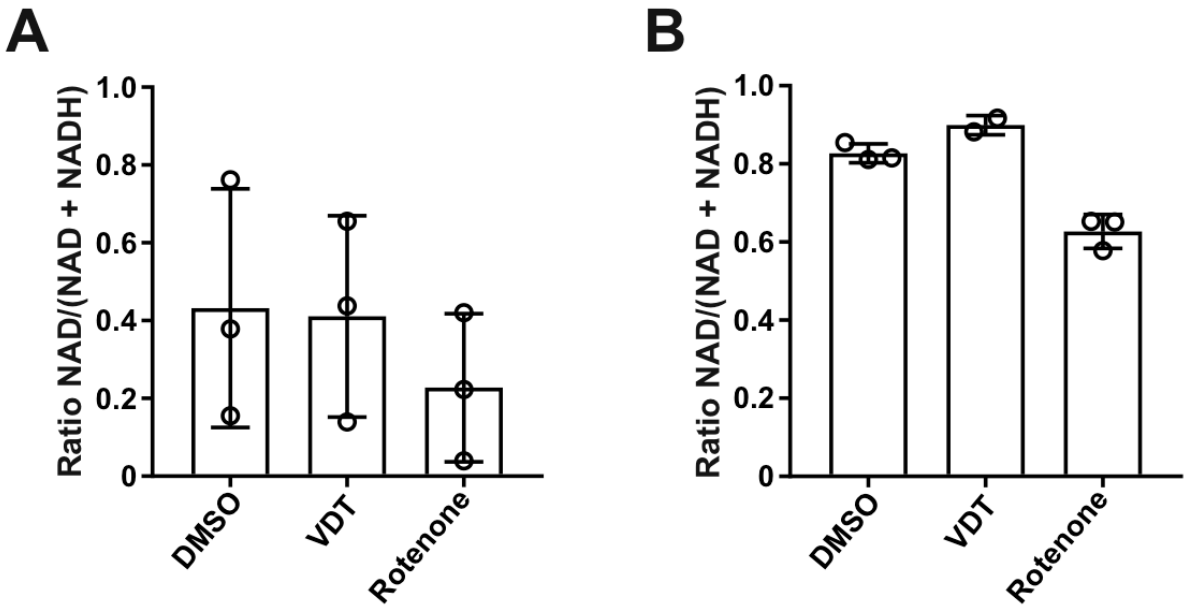

Suppl. Figure 4: VDT does not alter the NAD/NADH ratio

Besides mitoribosomal proteins other mitochondrial candidate proteins of the tricarboxylic acid (TCA, Krebs) cycle (such as PDHA1, PDHB, SUCLG1, SUCLG2, and IDH2; listed in Table 1) might be targeted by VDT. Since all these enzymes require NAD^+ and produce NADH + H^+ the NAD/NADH ratio was measured by (A) a fluorometric assay and (B) high-performance liquid chromatography. (A) Ramos cells were treated with DMSO, 10 µM VDT or 10 µM Rotenone for 30 min at 37 °C with 5% CO_2, harvested and lysed for 15 min at 37 °C. After centrifugation of the lysate, the supernatant was used for the assay, then the fluorescence signals were measured at Ex/Em 540/590 nm. Shown is the Ratio of NAD/(NAD + NADH) for three independent triplicates. (B) Ramos cells were treated with DMSO, 10 µM VDT or 10 µM Rotenone for 30 min at 37 °C with 5% CO_2, harvested and the NAD/(NAD + NADH) ratio determined by high-performance liquid chromatography as described [2].

Supplemental Materials and Methods

Thermal proteome profiling (TPP)

TPP except MS analysis and data analysis for protein identification and quantification was performed according to Franken et al. [3].

Compound treatment

Ramos cells were expanded as described in "Cell lines and cell culture" to 2.4 x 10^8 cells and treated (0.5 h, 37 °C, 5% CO_2) with VDT (10 µM) or vehicle (DMSO) at a density of 1.33 x 10^6 cells/ml. Cells were collected, washed with PBS and pelleted by centrifugation.

Temperature treatment

The two pellets of cells treated with VDT or vehicle were resuspended each in 2 ml of ice-cold PBS supplemented with protease inhibitor cocktail (cOmplete ULTRA EDTA-free Protease Inhibitor Cocktail Tablets, Roche) (PBS+PI) and 100 µl aliquots in ten PCR tube strips (each 2x VDT, 2x vehicle) were temperature treated (DNA Engine Tetrad 2, lid at 70 °C; 7 min pre-incubation and 3 min post-incubation at room temperature in a metal heating block) for 3 min at different temperatures (36.5, 41.2, 44.0, 47.1, 49.8, 53.3, 56.0, 59.2, 64.0, and 67.0 °C). Half of the VDT or vehicle samples were supplemented with 10% w/v NP40 (0.4% w/v final) for solubilization of membrane proteins [4, 5], the other half with water and shock frozen in liquid nitrogen and stored at -80 °C or processed directly.

The soluble protein fraction was obtained after cell lysis by means of four freeze/thaw cycles in liquid nitrogen and subsequent centrifugation (20000 g, 30 min, 4 °C). Protein concentrations were determined (Pierce 660 nm Protein-Assay, Thermo Scientific), and the samples were shock frozen in liquid nitrogen and stored at -80 °C.

As two independent biological replicates were analyzed, the procedure was repeated. In total, 80 samples [2 (replicates) x 2 (+/- VDT) x 2 (+/- NP40) x 10 (temperatures)] were obtained.

In-gel digestion, tandem mass tag (TMT) labeling and high pH reversed-phase peptide fractionation

Within each of the eight sample sets (ten temperatures), the same volume was used for further analysis, which was calculated from the nearly constant protein concentration of the three lowest temperature samples per sample set to contain 12.5 µg. Proteins were dried (SpeedVac concentrator), incubated (30 min, 50 °C, 700 rpm) with SDS sample buffer (25 mM DTT, 7.5% glycerol, 3% SDS, 37.5 mM Tris/HCl pH 7.0, 0.005% bromophenol blue) and carbamidomethylated using iodoacetamide (100 mM final, 30 min, room temperature, in the dark). The samples were subjected to short SDS-PAGE (30 min at 50 V; 26-well 4 - 12% Bis-Tris polyacrylamide gels, Novex NuPAGE, Thermo Scientific; MOPS running buffer), Coomassie Brilliant Blue stained, and gel lanes were cut out and stored at -80 °C. In-gel digestion was performed from destained gel lanes using 0.28 µg Trypsin/Lys-C-Mix (Mass Spec Grade, Promega, V5073) in 74 µl 5 mM TEAB per gel lane for 3.5 h at 37 °C followed by collection of the supernatants and overnight digestion at 37 °C using 0.42 µg Trypsin/Lys-C-Mix in 21 µl 5 mM TEAB per gel lane. Peptides from corresponding supernatants of the gel lanes after digestion (3.5 h and overnight) and extraction [1% formic acid (FA), 60% acetonitrile (ACN) in 1% FA, and finally 100% CAN] were combined. 40 vol% (5 µg peptides on average for the lowest three temperatures assuming quantitative yield), respectively, of the collected supernatants containing the extracted peptides were dried (SpeedVac).

The dried peptides of each sample were reconstituted in 5 µl 180 mM TEAB in 10% (v/v) ACN. TMT labeling (10-plex isobaric label reagent set, Thermo Scientific, #90110) was performed using 39 µg of each label in 2 µl ACN (36.5 °C/TMT 131, 41.2 °C/130C, 44.0 °C/130N, 47.1 °C/129C, 49.8 °C/129N, 53.3 °C/128C, 56.0 °C/128N, 59.2 °C/127C, 64.0 °C/127N, 67.0 °C/126) for 1 h at room temperature followed by quenching (0.8 µl 5% hydroxylamine, >15 min, room temperature). The combined TMT labeled peptide samples of each sample set were shock frozen in liquid nitrogen, dried (SpeedVac), and stored at -80 °C. Offline peptide pre-fractionation was performed using a commercially available kit (Pierce High pH Reversed-Phase Peptide Fractionation Kit, #84868, Thermo scientific)

according to the manufacturer's specifications for TMT-labeled peptides. The resulting eight fractions (300 µl each) per sample set were stored at -80 °C.

Mass spectrometric (MS) measurement

28.4 µl (assuming 0.3 µg peptides) per fraction were dried (SpeedVac), reconstituted in 17 µl 0.1% trifluoroacetic acid (TFA) and 15 µl were subjected to MS measurement employing the following setup and conditions. Peptide separation was performed on a liquid chromatography system (RSLC, Ultimate 3000, Thermo Fisher). First, peptides were concentrated on a trap column (Acclaim PepMap100, 3 µm C18 particle size, 100 Å pore size, 75 µm inner diameter, 2 cm length, Thermo Fisher Scientific, Dreieich, Germany) using a flow rate of 6 µl/min for 10 min with 0.1% TFA as mobile phase. Then, the peptides were separated on the analytical column (Acclaim PepMapRSLC, 2 µm C18 particle size, 100 Å pore size, 75 µm inner diameter, 25 cm length, Thermo Fisher Scientific, Dreieich, Germany) heated to 60 °C using a flow rate of 300 nl/min and a 2 h gradient from 4 to 40% solvent B (solvent A: 0.1% (v/v) formic acid in water; solvent B: 0.1% (v/v) formic acid, 84% (v/v) ACN in water).

Peptides were sprayed via a nano-source ESI interface equipped with distally coated SilicaTip emitters (New Objective, Woburn, MA, USA) into the online coupled Orbitrap Fusion Lumos Tribrid mass spectrometer (Thermo Fisher Scientific, Dreieich, Germany; Software: Xcalibur 4.3.73.11, SII 1.5.0.10747, Orbitrap Fusion Lumos 3.3.2782.28) operated in positive mode. Precursor mass spectra were recorded in the orbitrap analyzer within a mass range of 375 - 1500 m/z and a resolution of 120000. MS^2 spectra were recorded with scan rate 'turbo' (125000 Da/s) in the linear ion trap. Precursors for MS^3 spectra were selected in a mass range of 400 - 1200 m/z with a 2 m/z isolation window using isobaric tag exclusion filtering with TMT as the reagent tag type as well as synchronous precursor selection (SPS, maximum 10 notches). The precursors were fragmented via higher-energy collisional dissociation and MS^3 spectra were recorded in the orbitrap analyzer within a mass range of 100 - 500 m/z and a resolution of 50000.

Mass spectrometric data analysis, protein identification and quantification

Mass spectrometric data were processed with MaxQuant version 1.6.6.0 (Max Planck Institute for Biochemistry, Planegg, Germany) using standard parameters for protein identification (peptide and protein FDR of 1%) and quantification based on 74416 Homo sapiens protein entries downloaded from the UniProtKB on 19th June 2019 and tryptic cleavage specificity (≤ 3 missed cleavage sites). Methionine oxidation and N-terminal acetylation as well as a carbamidomethylation at cysteine residues were considered as variable and fixed modifications, respectively. For first and second search precursor mass tolerance was set to 20 ppm and 4.5 ppm, respectively. Mass tolerances for fragment spectra were 20 ppm. Each of the eight experiments (sample sets) was defined with eight fractions. The settings "Label-free quantification" and "Match between runs" were disabled and the following settings were explicitly enabled: (i) As group-specific parameters, "Reporter ion MS3 with 10plex TMT" was chosen without correction factors and a reporter mass tolerance of 0.003 Da. (ii) As global parameters, under "Protein quantification", "Discard unmodified counterpart peptides" and "Advanced ratio estimation" and under "Tables", "Write ms3Scans table" were enabled.

Protein melting curve analysis

For normalization of the 4424 identified proteins, the reporter intensity for each protein at the respective temperature point and experiment was multiplied by the ratio of the fitted intensity (fitting procedure described below) at this temperature point and the median of all valid protein reporter intensity values at this temperature point within this experiment.

The above described fitted intensity $f(T)$ at each temperature point T was obtained by a least square fit to the mean – over the 8 experiments – of the median reporter intensity values at this temperature point using the logistic function

$$f(T) = f_{min} + (f_{max} - f_{min}) \,/\, \{ 1 + \exp \{ b \times [\log_{10}(T) - \log_{10}(T_m)] \} \},$$

with the fitting parameters f_{min} as the lower limit, f_{max} as the upper limit, b as a slope parameter, and T_m as a melting point parameter.

The normalized reporter intensities were then used as input for melting curve (TPP-TR) analysis using the TPP analysis package v3.13.3 in “R” v3.6.1 [3, 6]. In accordance with a previous study [4], the presence of detergent during extraction after cell heating generally did not affect protein T_m values. Therefore, the four VDT treated samples (+/- NP40) were compared to the four vehicle controls (+/- NP40) using the NPARC algorithm within the TPP software package resulting in adjusted p-values for each protein. The negative decadic logarithm of this adjusted NPARC p-value, -lg(adj. p, NPARC), was plotted against the difference of the means of the melting points, ΔT_m, of the VDT treated samples and the vehicle controls for each protein (Figure 7A). Proteins with an incomplete set of valid melting point values were omitted from the analysis resulting in a set of 2688 proteins comprising a complete set of valid melting point values. Four proteins (UniProt identifiers K7ES61, P11177, Q13084, Q9BYD1) had a zero value software output for their adjusted NPARC p-values and were manually assigned with an arbitrary reasonable value of -lg(adj. p, NPARC) = 15 (the lowest non-zero adjusted NPARC p-value was 3.9×10^{-13}, i.e., -lg(adj. p, NPARC) = 12.4) after checking the correctness of data and melting curves. Mitoribosomal proteins (red circles in Figure 7A) were marked according to their annotation in the UniProt database.

Proteins with -lg(adj. p, NPARC) >3.85 (and R^2 >0.8 for each melting curve fit) were regarded as significantly stabilized (ΔT_m >0: 23 proteins) or destabilized (ΔT_m <0: 43 proteins) caused by direct or indirect effects of VDT treatment and were further analyzed for protein-protein association networks using the online string data base [7].

Measurement of tubulin polymerization velocity (see Suppl. Fig. 2A,B)

Measurements of tubulin polymerization velocity under treatment with VDT and suitable positive controls were performed using the *OD based tubulin polymerization assay kit* (Cytoskeleton, Inc., Denver, CO, USA; #BK006P) in porcine mitochondria according to manufacturer's instructions. The kit is based on the observation that polymerization of monomeric tubulin is accompanied by an increase in absorption at 340 nm, which can be used as a measure of the underlying reaction. The velocity of polymerization has been calculated using the formulas provided by the manufacturer. Paclitaxel stabilizes tubulin and

therefore increases the polymerization rate, whereas vinblastine destabilizes tubulin and consequently reduces the polymerization rate and were used as controls accordingly.

Detection of *in situ* tubulin polymerization via immunoblotting (see Suppl. Fig. 2C)

To trace a possible shift in the ratio between soluble (cytosolic) and polymerized (cytoskeletal) tubulin an immunoblot-based method was used similar to that described by Hood et al. [8]. Briefly, Ramos cells were treated with the test compounds, harvested by centrifugation (1000 g, 5 min) and subsequently lysed in a hypotonic lysis buffer [1 mM $MgCl_2$, 2 mM EGTA, 1% NP40, 2 mM phenylmethylsulfonyl fluoride, 1 µg/ml aprotinin, 2 µg/ml pepstatin, and 20 mM Tris-HCl (pH 6.8)] for 5 min at 37 °C. Subsequently, soluble tubulin was separated from the polymerized tubulin by centrifugation (10 min, 18000 g). After dissolving the resulting pellet (containing polymerized tubulin) in 20 µl sample buffer (8 M urea, 4% 3-(3-cholamidopropyl)dimethylammonio)-1-propanesulfonic acid, 3 M thiourea, and 40 mM DTT) and adding loading buffer to all samples, SDS-PAGE and immunoblot analyses with anti-tubulin (TUBA4A; Sigma, #T5168) and anti-vinculin (Sigma, #V9131) were performed as described above.

Detection of NAD/NADH ratio by enzymatic assay (see Suppl. Fig. 4A)

To measure the NAD+/NADH ratio in cell extracts the NAD/NADH assay kit from Abcam (#176723) was performed according to manufacturer's instructions. Briefly, Ramos cells (10^7 cells/ treatment) were treated with the test compounds (DMSO, 10 µM Viriditoxin and Rotenone as a positive control) for 30 min at 37 °C with 5% CO_2, harvested by centrifugation (1500 rpm, 5 min), subsequently lysed in 100 µl lysis buffer and incubated for 15 min at 37 °C. The lysate was centrifuged at 1500 rpm for 5 min and the supernatant was used for the assay. Standard preparation and assay procedure were conducted according to manufacturer's instructions, the fluorescence increase was measured on a microplate reader at Ex/Em 540/590 nm. The sample calculations were performed according to manufacturer's instructions. The ratio of NAD^+ to NADH in the test samples was calculated and set in

relation to the total of NAD^+/NADH (NAD^+/(NAD^+ + NADH)). Each experiment was performed in triplicates.

Detection of NAD/NADH ratio by high-performance liquid chromatography (see Suppl. Fig. 4B)

The detection of NAD+/NADH ratio was further elucidated by mass spectrometry. Therefore, Ramos cells (10^7 cells/treatment) were treated with the test compounds (DMSO, 10 µM VDT and Rotenone as positive control) for 30 min at 37 °C with 5% CO_2, harvested by centrifugation (1500 rpm, 5min) and the NAD/NADH ratio determined by high-performance liquid chromatography as described [2].

Supplemental References

1. Nicoletti I, Migliorati G, Pagliacci MC, Grignani F, Riccardi C. A rapid and simple method for measuring thymocyte apotosis by propidium iodide staining and flow cytometry. J Immunol Methods 1991;139:271-9.
2. Smolenski R, Lachno D, Ledingham S, Yacoub M. Determination of sixteen nucleotides, nucleosides and bases using high-performance liquid chromatography and its application to the study of purine metabolism in hearts for transplantation. J Chromatogr 1990;527:414-20.
3. Franken H, Mathieson T, Childs D, Sweetman GMA, Werner T, Tögel I, et al. Thermal proteome profiling for unbiased identification of direct and indirect drug targets using multiplexed quantitative mass spectrometry. Nat Protoc 2015;10:1567-93.
4. Reinhard FB, Eberhard D, Werner T, Franken H, Childs D, Doce C, et al. Thermal proteome profiling monitors ligand interactions with cellular membrane proteins. Nat Methods 2015;12:1129-31.
5. Huber KV, Olek KM, Muller AC, Tan CS, Bennett KL, Colinge J, et al. Proteome-wide drug and metabolite interaction mapping by thermal-stability profiling. Nat Methods 2015;12:1055-7.
6. Childs D, Bach K, Franken H, Anders S, Kurzawa N, Bantscheff M, et al. Nonparametric Analysis of Thermal Proteome Profiles Reveals Novel Drug-binding Proteins. Mol Cell Proteomics 2019;18:2506-15.
7. Szklarczyk D, Gable AL, Lyon D, Junge A, Wyder S, Huerta-Cepas J, et al. STRING v11: protein-protein association networks with increased coverage, supporting functional discovery in genome-wide experimental datasets. Nucleic Acids Res 2019;47:D607-D13.
8. Hood KA, West LM, Rouwe B, Northcote PT, Berridge MV, Wakefield SJ, et al. Peloruside A, a Novel Antimitotic Agent with Paclitaxel-like Microtubule-stabilizing Activity. Cancer Res 2002;62:3356-60.
