# Supplementary material for: The mycotoxin viriditoxin induces leukemia- and lymphoma-specific apoptosis by targeting mitochondrial metabolism
Source: Cell Death Dis. 2022 Nov 8;13(11):938. doi: 10.1038/s41419-022-05356-w (PMC9643474; doi:10.1038/s41419-022-05356-w)

Original Western Blots

(all western blots were scanned via LI-COR Odyssey® imaging system)

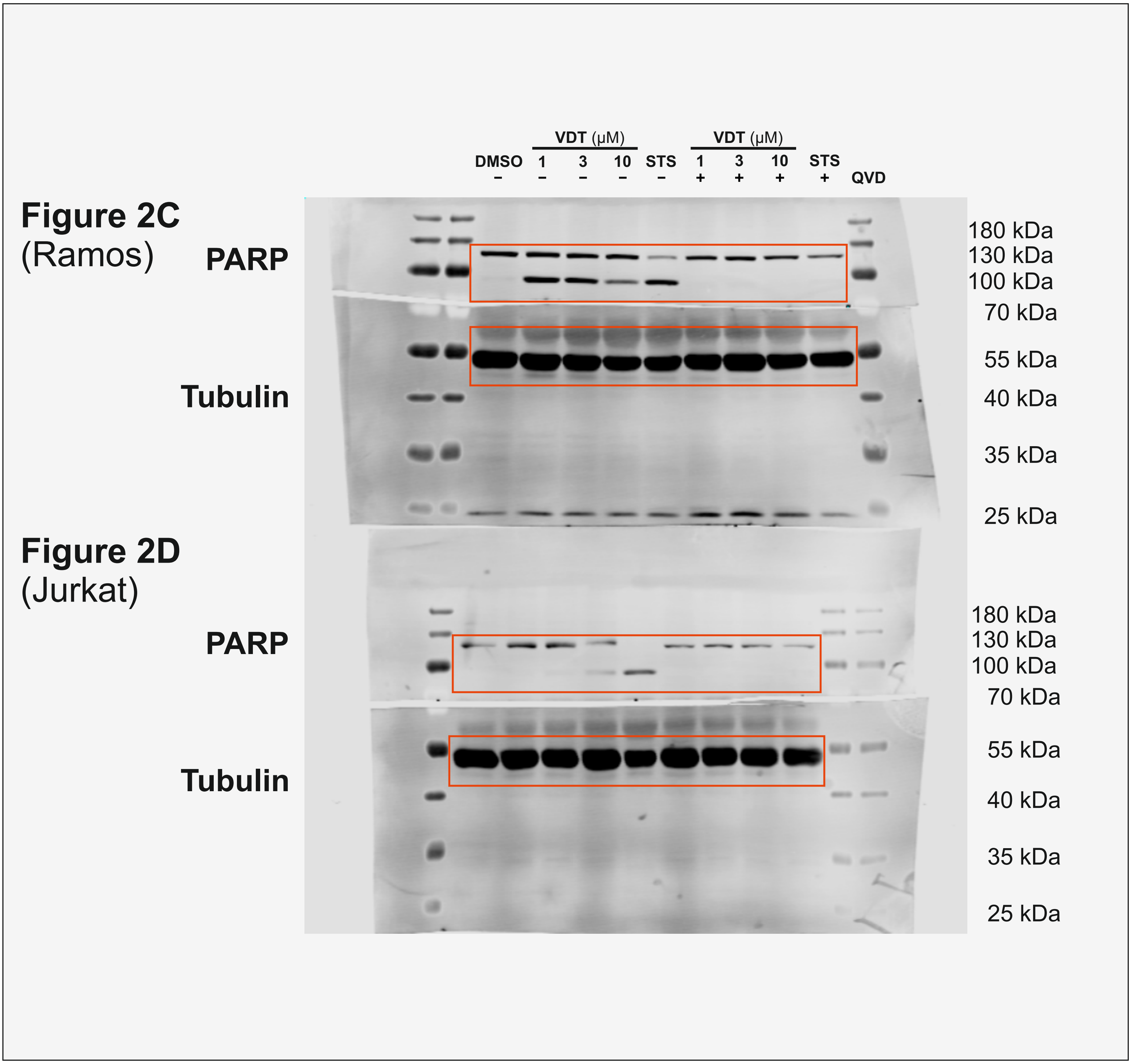

Figure 4B

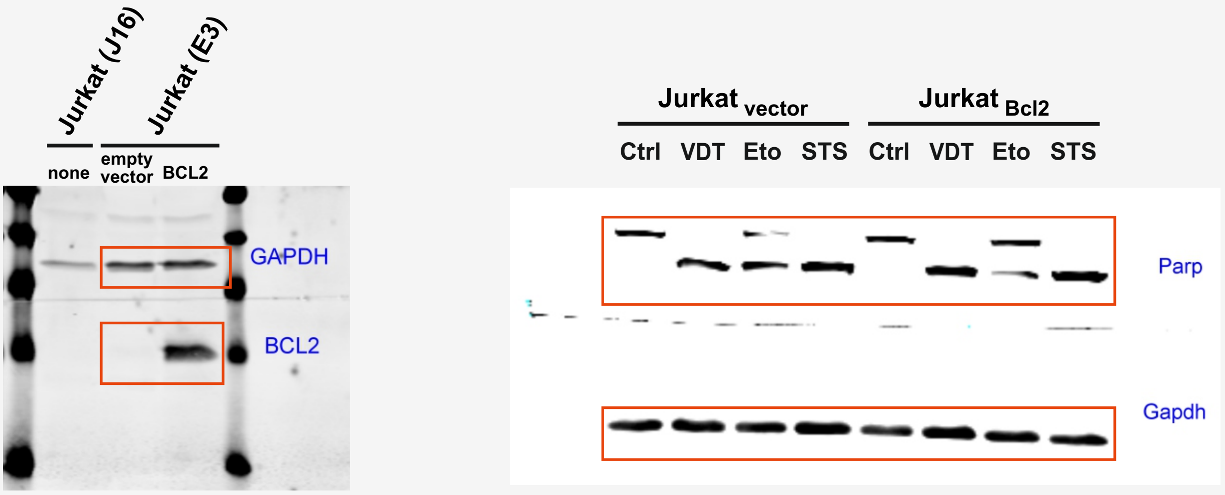

Figure 4E

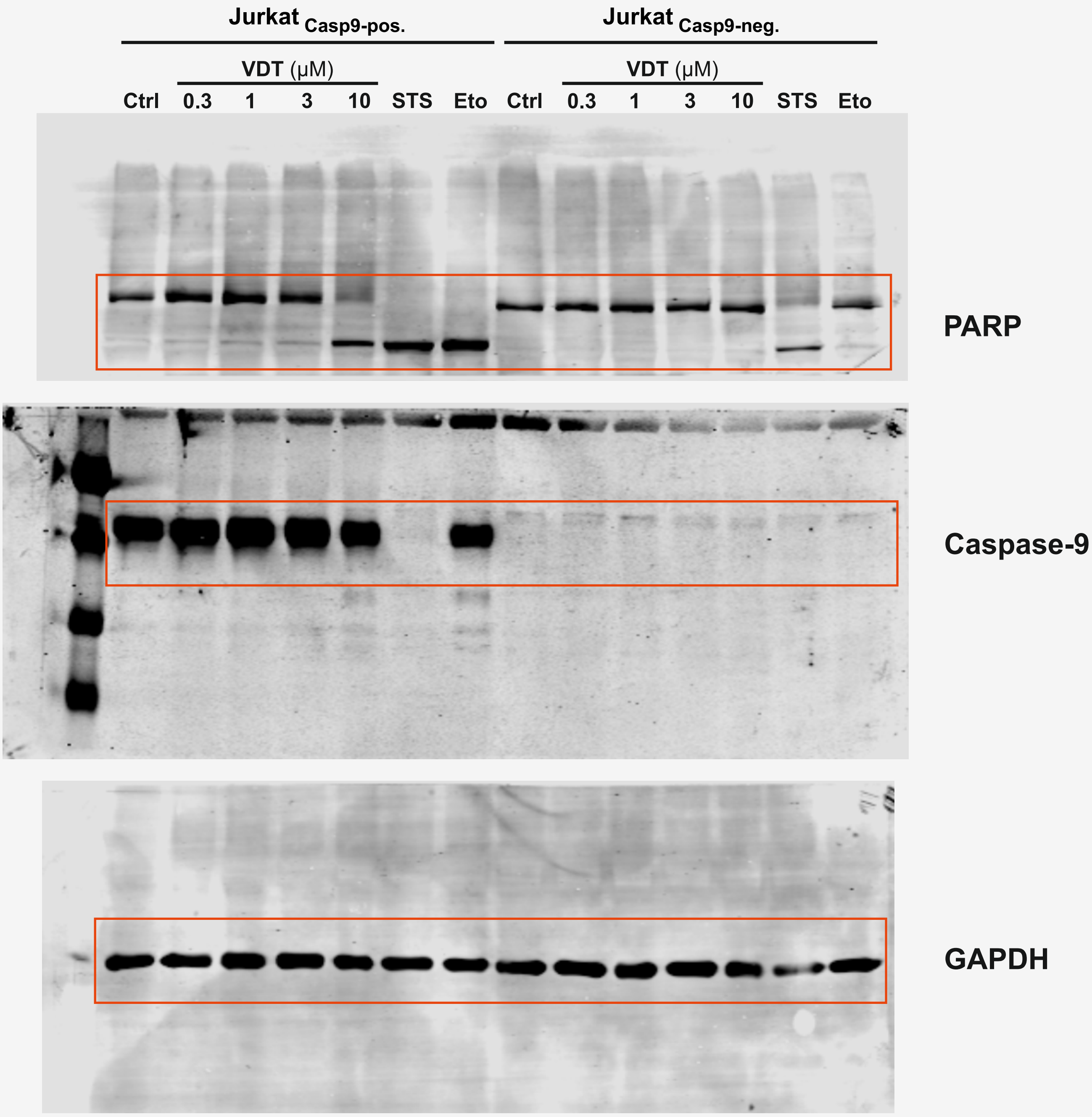

Figure 4G

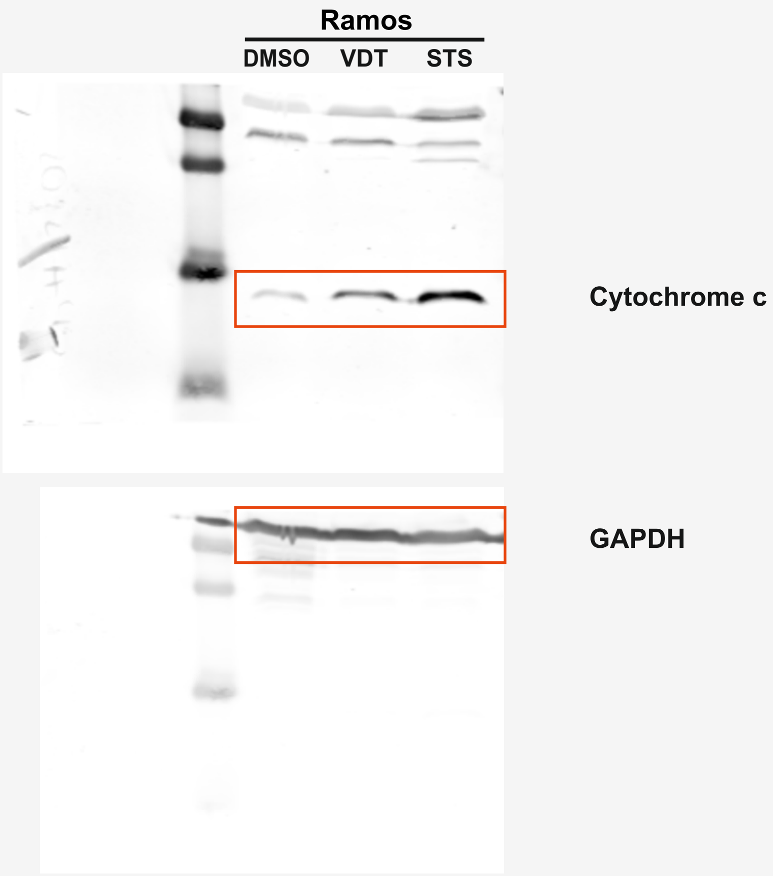

Figure 5A
left panel)

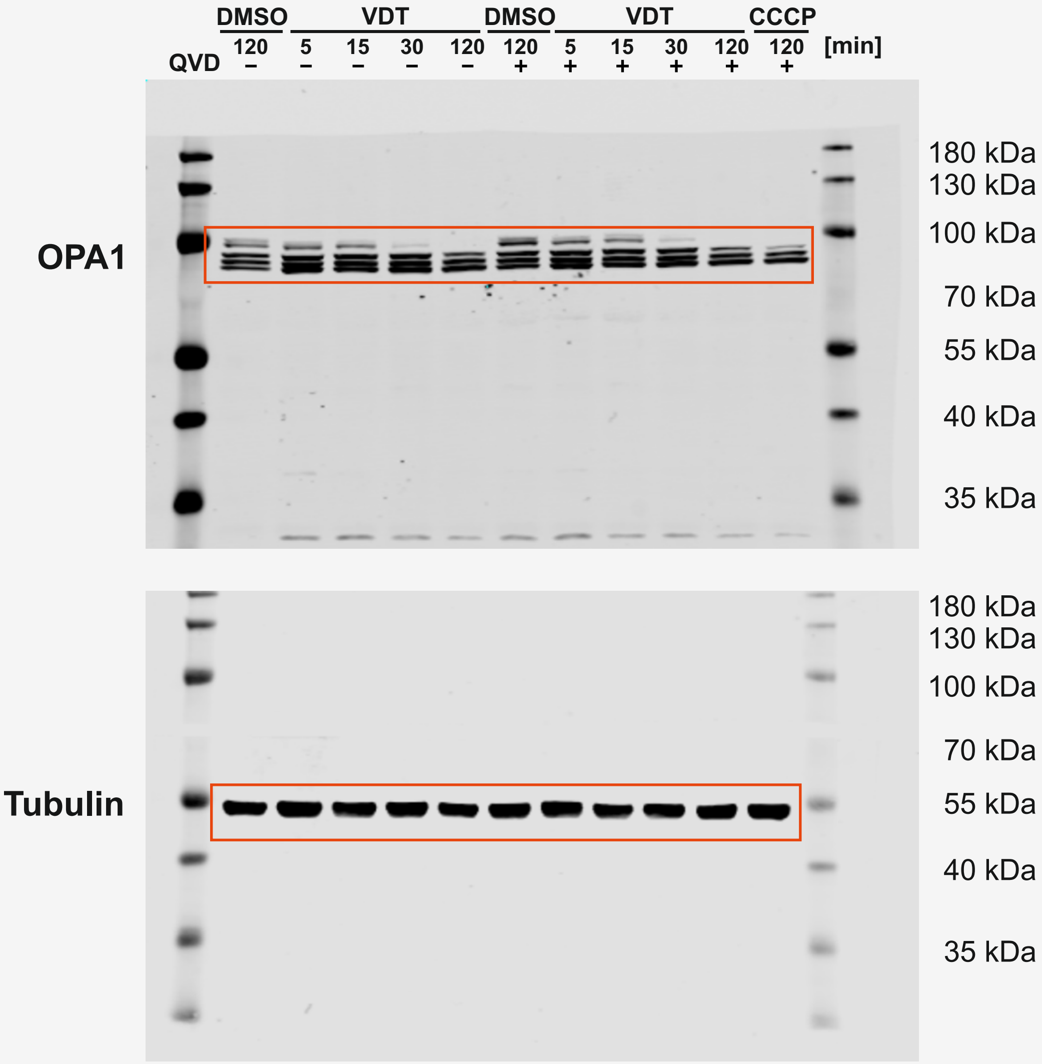

Figure 5A
right panel)

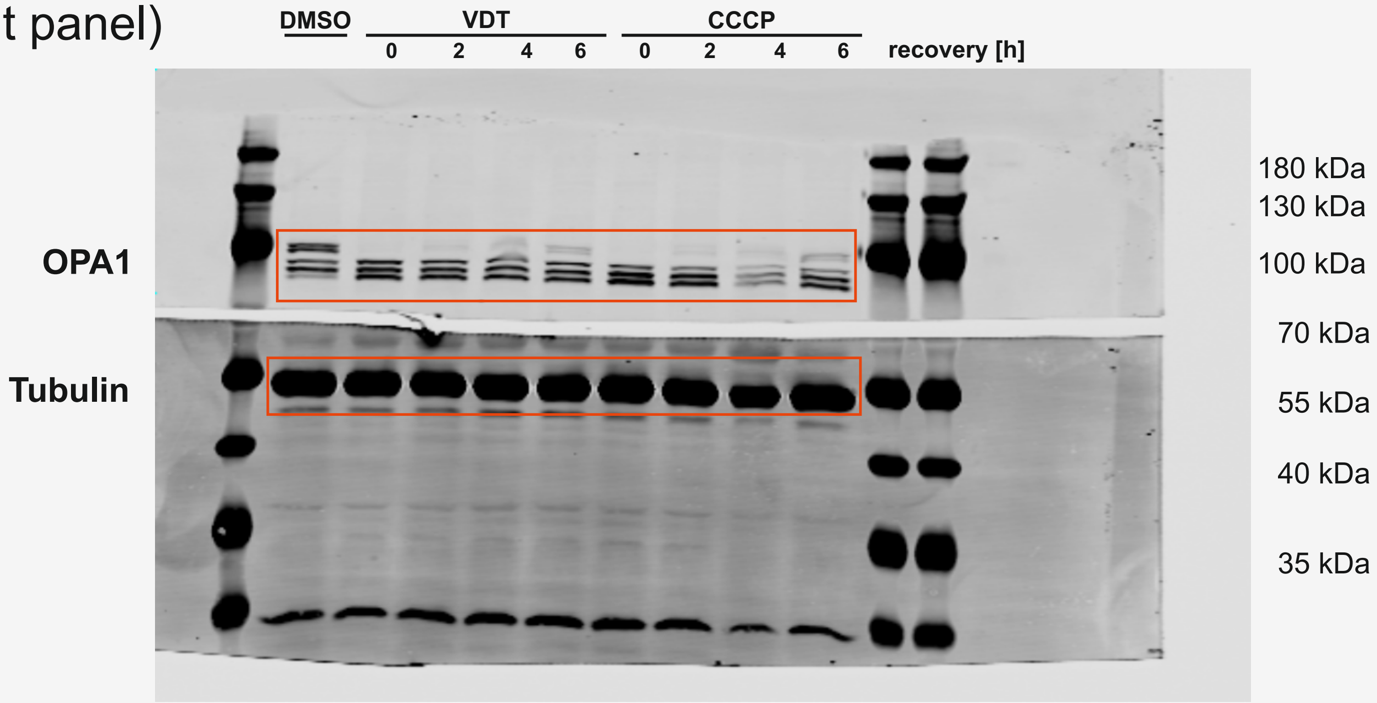

Figure 5F

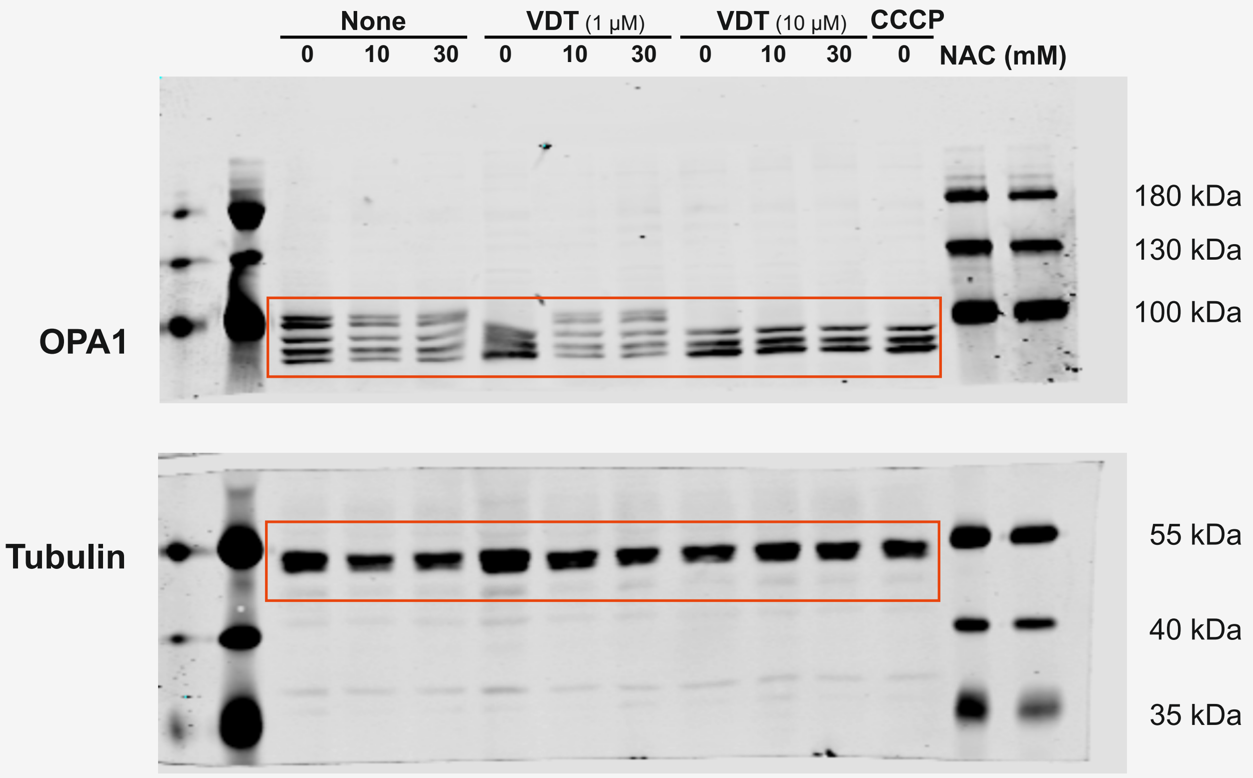

Figure 6D

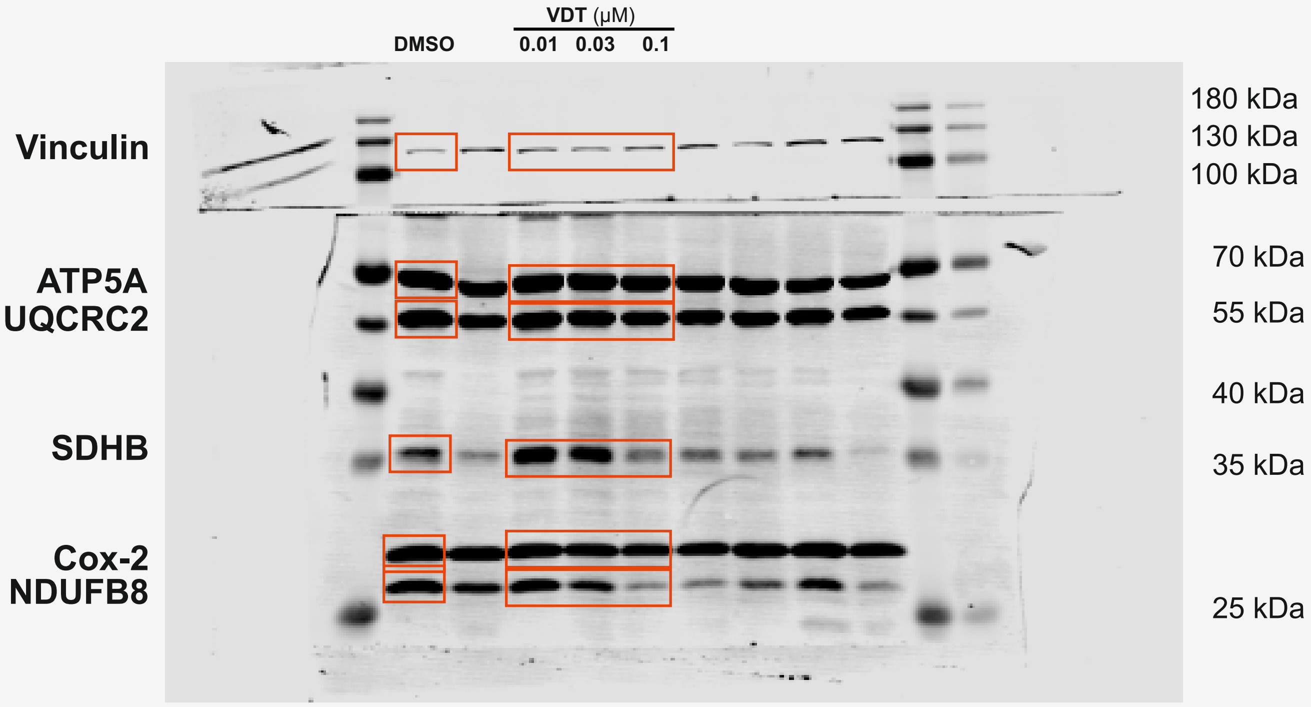

Figure 7C

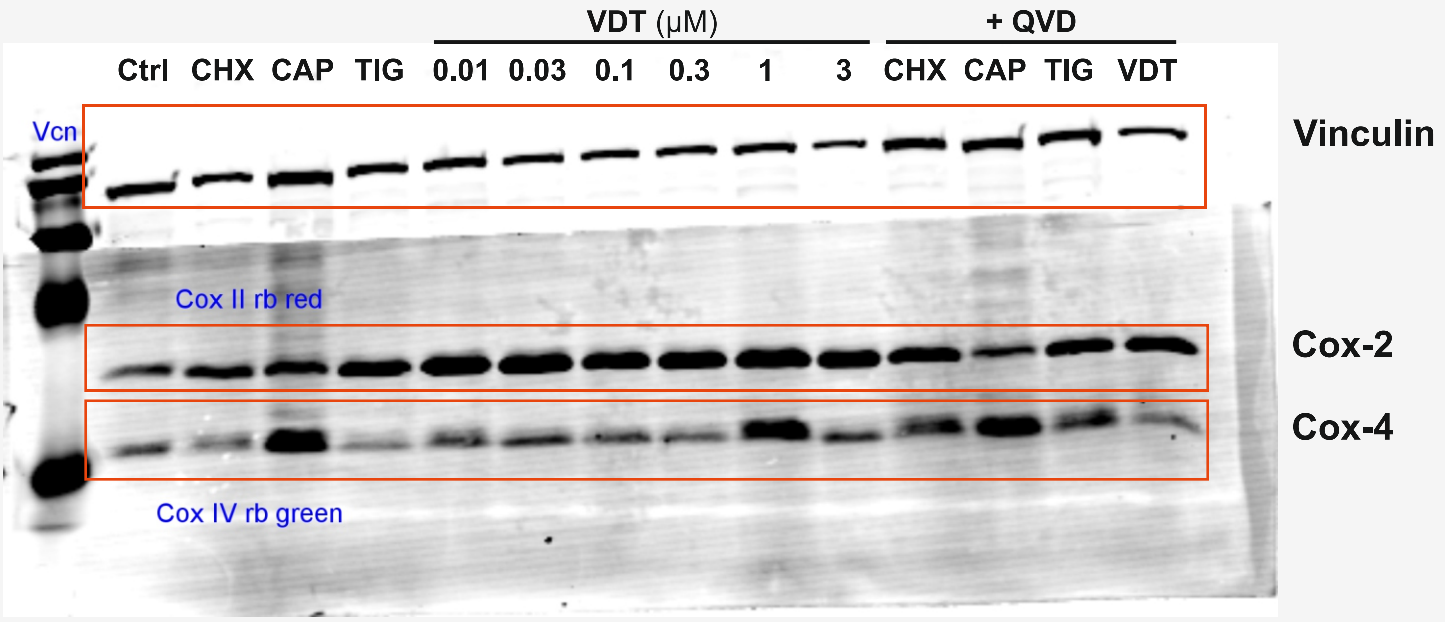
VDT (μM)
+ QVD
Ctrl
CHX
CAP
TIG
0.01
0.03
0.1
0.3
1
3
CHX
CAP
TIG
VDT
Vcn
Vinculin
Cox II rb red
Cox-2
Cox-4
Cox IV rb green

Suppl. Figure 2C

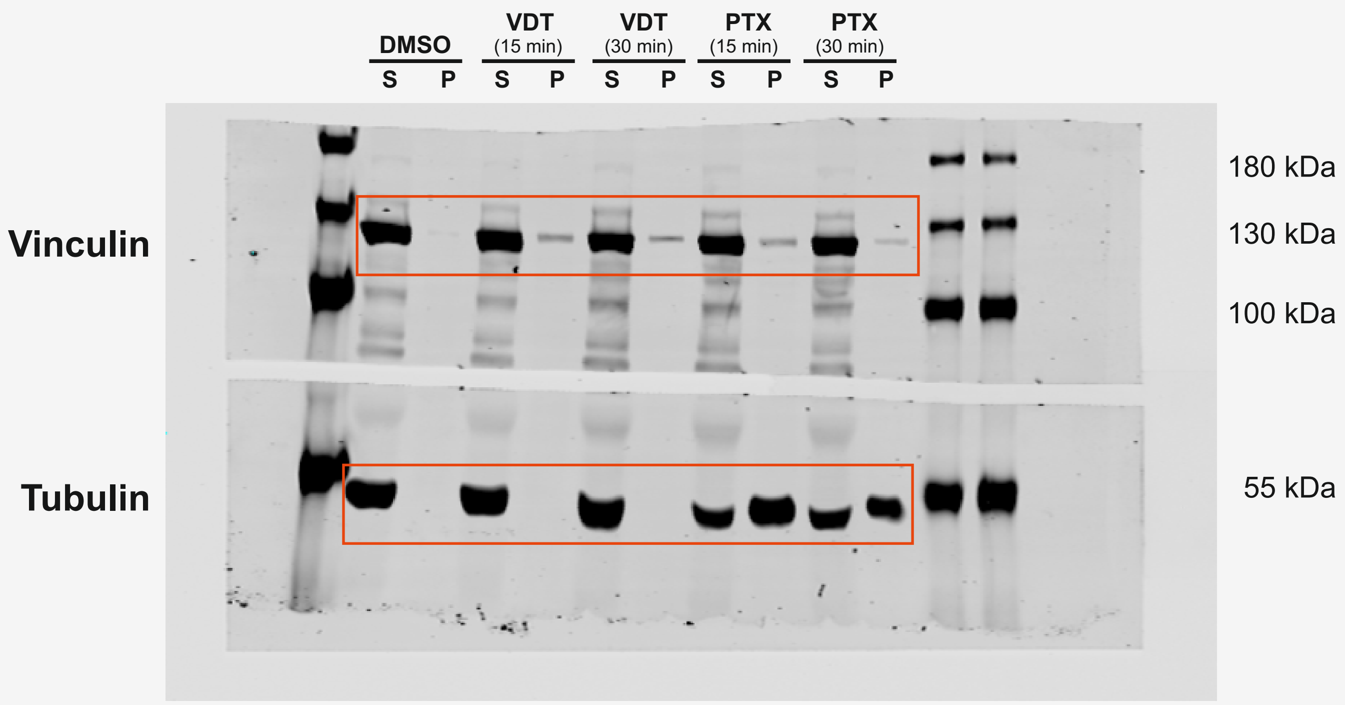

Suppl. Figure 3B

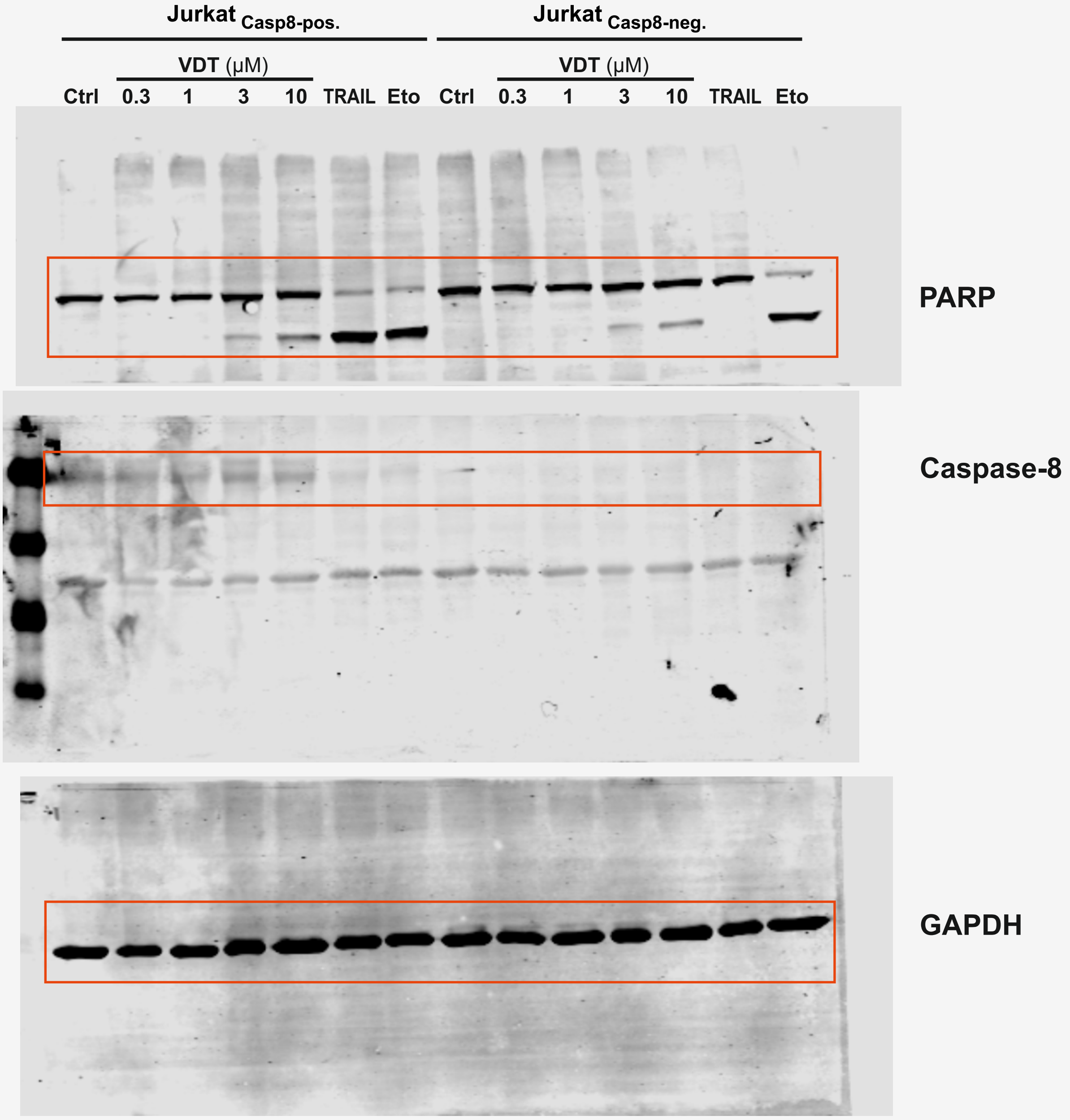

Supplement: Supplementary file 2 — Original Data File [file 41419_2022_5356_MOESM2_ESM.pdf]
