# Supplementary material for: The mycotoxin viriditoxin induces leukemia- and lymphoma-specific apoptosis by targeting mitochondrial metabolism
Source: Cell Death Dis. 2022 Nov 8;13(11):938. doi: 10.1038/s41419-022-05356-w (PMC9643474; doi:10.1038/s41419-022-05356-w)
Supplement: Supplementary file 3 — Author confirmation concerning changes of author list (see your E-mail from October 5th 2022) [file 41419_2022_5356_MOESM3_ESM.pdf]

Agreement from all authors concerning altered author list (CDDIS-22-1280RR)

Listed in order of the author list in the manuscript

Fabian Stuhldreier

Von: Fabian Stuhldreier <fabianstuhldreier@gmail.com>
Betreff: Aw: Fwd: CDDIS-22-1280RR Initial Quality Check
Datum: 8. Oktober 2022 um 00:23:35 MESZ
An: Sebastian Wesselborg <sebastian.wesselborg@uni-duesseldorf.de>

Dear Sebastian,

I fully agree with the modified list of authors.

With kind regards Fabian Stuhldreier

Am 06.10.2022 um 11:07 schrieb Sebastian Wesselborg:
Dear colleagues,
Cell Death & Disease has noted that the author list from our first submission differs from the resubmission since we included additional authors (no deletions) due to their valuable contributions for the rebuttal. The following authors were added: Sanil Bhatia, Julian Schliehe-Diecks, Bodo Levkau, Philipp Wollnitzke, Andreas Reichert, Marcel Zimmermann, and Nicole Teusch.

Therefore, I need the confirmation from all authors that you agree to these changes. **Please send me this confirmation per e-mail so I can upload it for Cell Death & Disease.**

With best regards
Sebastian

****
Prof. Sebastian Wesselborg, PhD
Director, Institute for Molecular Medicine I
School of Medicine
University of Duesseldorf
Universitätsstr. 1; Bld. 22.03
D-40225 Duesseldorf
GERMANY
Tel: +49-(0)211-81 12722
+49-(0)172-1079 468
E-Mail: sebastian.wesselborg@uni-duesseldorf.de
https://www.uniklinik-duesseldorf.de/patienten-besucher/klinikeninstitutezentren/institut-fuer-molekulare-medizin-i

Laura Schmitt

Von: <Laura.Schmitt@med.uni-duesseldorf.de>
Betreff: AW: CDDIS-22-1280RR Initial Quality Check
Datum: 6. Oktober 2022 um 11:14:32 MESZ
An: <sebastian.wesselborg@uni-duesseldorf.de>

I agree to these changes.
Best

Laura Schmitt
Doktorandin, Institut für Molekulare Medizin I

Universitätsklinikum Düsseldorf
Gebäude 22.03, Ebene 01, Raum 26
Universitätsstraße 1
40225 Düsseldorf

Mail: laura.schmitt@med.uni-duesseldorf.de
Tel: +49 211 81-13554

Thomas Lenz

Von: "Thomas Lenz" <thomas.lenz@hhu.de>
Betreff: AW: CDDIS-22-1280RR Initial Quality Check
Datum: 6. Oktober 2022 um 11:12:06 MESZ
An: "'Sebastian Wesselborg'" <sebastian.wesselborg@uni-duesseldorf.de>

Dear Sebastian,

I agree to the changes.

Best,
Thomas

Ilka Hinxlage

Von: "Ilka Hinxlage" <Ilka.Hinxlage@hhu.de>
Betreff: AW: URGENT !!! Wtr: CDDIS-22-1280RR Initial Quality Check
Datum: 10. Oktober 2022 um 09:21:44 MESZ
An: "'Sebastian Wesselborg'" <sebastian.wesselborg@uni-duesseldorf.de>

Guten Morgen Sebastian,

hier von mir auch das statement:

Herewith, I confirm that I fully comply with the changes concerning the final author list.

Dir einen guten Start in die Woche!

Ilka

--

Ilka Hinxlage
Doktorandin, Institut für Molekulare Medizin I

Universitätsklinikum Düsseldorf
Gebäude 22.03, Ebene 01, Raum 26
40225 Düsseldorf

Tel: +49 211 81-13554
e-Mail: ilka.hinxlage@hhu.de

Marcel Zimmermann

Von: "Zimmermann, Marcel" <Marcel.Zimmermann.2@uni-duesseldorf.de>
Betreff: AW: URGENT !!! Wtr: CDDIS-22-1280RR Initial Quality Check
Datum: 14. Oktober 2022 um 10:25:38 MESZ
An: Sebastian Wesselborg <sebastian.wesselborg@uni-duesseldorf.de>

Dear Sebastian,

I hereby fully agree to the final author list and order and all changes that have been made.

Please forward this email to the Journal.

Sincerly

Marcel Zimmermann

Philipp Wollnitzke

Von: <Philipp.Wollnitzke@med.uni-duesseldorf.de>
Betreff: AW: URGENT !!! Wtr: CDDIS-22-1280RR Initial Quality Check
Datum: 10. Oktober 2022 um 07:33:34 MESZ
An: <sebastian.wesselborg@uni-duesseldorf.de>

Dear Sebastian,

thank you for considering me as co-author. Herewith, I confirm that I fully comply with the changes concerning the final author list.

Best regards,

Philipp

Julian Schliehe-Dieckss

Von: <Julian.Schliehe-Dieckss@med.uni-duesseldorf.de>
Betreff: AW: CDDIS-22-1280RR Initial Quality Check
Datum: 6. Oktober 2022 um 11:11:24 MESZ
An: <sebastian.wesselborg@uni-duesseldorf.de>

Dear Prof. Wesselborg,

I agree with the author changes.

BR,

Julian Schliehe-Dieckss

Yang Liu

Von: 刘洋 <liuyangthink@163.com>
Betreff: Re:URGENT !!! Wtr: CDDIS-22-1280RR Initial Quality Check
Datum: 12. Oktober 2022 um 15:54:09 MESZ
An: "Sebastian Wesselborg" <sebastian.wesselborg@uni-duesseldorf.de>

Dear Prof. Wesselborg,

really sorry to reply to your email so late. I agree with the changes with the addition of other authors!

Best regards!

Yang Liu

Paul Jäger

on: <PaulSebastian.Jaeger@med.uni-duesseldorf.de>
Betreff: AW: URGENT !!! Wtr: CDDIS-22-1280RR Initial Quality Check
Datum: 9. Oktober 2022 um 20:05:15 MESZ
An: <sebastian.wesselborg@uni-duesseldorf.de>

Dear Sebastian,

thank you very much.

Herewith, I confirm that I fully comply with the changes made concerning the final author list.

With kind regards
Paul Jäger

Stefanie Geyh

Von: <Stefanie.Geyh@med.uni-duesseldorf.de>
Betreff: AW: URGENT !!! Wtr: CDDIS-22-1280RR Initial Quality Check
Datum: 9. Oktober 2022 um 21:29:51 MESZ
An: <sebastian.wesselborg@uni-duesseldorf.de>

Dear Sebastian,

I apologize for the delayed reply, I just got back from an outside conference. Thanks for your efforts regarding the manuscript.

Herewith, I confirm that I fully comply with the changes concerning the final author list.

Kind regards,
Stefanie Geyh

Dr. rer. nat. Stefanie Geyh
Klinik f. Hämatologie, Onkologie u. Klinische Immunologie
Hämatologisches Forschungslabor
Universitätsklinikum Düsseldorf
Moorenstr. 5
Geb. 14.83.01.07
40225 Düsseldorf
Tel: +49-211-81-19606
Fax: +49-211-81-04296

Nicole Teusch

Von: "Prof. Dr. Nicole Teusch" <Nicole.Teusch@hhu.de>
Betreff: Aw: URGENT !!! Wtr: CDDIS-22-1280RR Initial Quality Check
Datum: 9. Oktober 2022 um 11:18:34 MESZ
An: Sebastian Wesselborg <sebastian.wesselborg@uni-duesseldorf.de>

Dear Sebastian,

I agree with and confirm my authorship in the mentioned manuscript.
Thank you for your consideration.

Kind regards
Nicole

Christoph Peter

Von: "Christoph Peter" <christoph.peter@uni-duesseldorf.de>
Betreff: AW: CDDIS-22-1280RR Initial Quality Check
Datum: 6. Oktober 2022 um 11:15:44 MESZ
An: "'Sebastian Wesselborg'" <sebastian.wesselborg@uni-duesseldorf.de>

Dear Sebastian,

I hereby confirm that I agree with these changes.

Best
Christoph

Christoph Peter, Ph.D.
Institute of Molecular Medicine
University Hospital Düsseldorf
Building 22.03
Universitätsstraße 1
40225 Düsseldorf
Germany

Ph.: +49 (0) 211 81-12196
E-mail: christoph.peter@uni-duesseldorf.de

Sanil Bhatia

Von: <Sanil.Bhatia@med.uni-duesseldorf.de>
Betreff: AW: CDDIS-22-1280RR Initial Quality Check
Datum: 6. Oktober 2022 um 11:56:57 MESZ
An: <sebastian.wesselborg@uni-duesseldorf.de>

Dear Sebastian,

I confirm the changes in the authorship concerning the manuscript entitled 'The mycotoxin viriditoxin induces leukemia- and lymphoma-specific apoptosis by targeting mitochondrial metabolism: CDDIS-22-1280RR'.

Best regards,
Sanil

Rainer Haas

Von: <Haas@med.uni-duesseldorf.de>
Betreff: AW: CDDIS-22-1280RR Initial Quality Check
Datum: 7. Oktober 2022 um 14:13:14 MESZ
An: <sebastian.wesselborg@uni-duesseldorf.de>, <Fabian.Stuhldreier@uni-duesseldorf.de>, <fabianstuhldreier@gmail.com>, <fabianstuhldreier@googlemail.com>, <Laura.Schmitt@med.uni-duesseldorf.de>, <Ilka.Hinxlage@hhu.de>, <Thomas.Lenz@uni-duesseldorf.de>, <Marcel.Zimmermann.2@hhu.de>, <Philipp.Wollnitzke@med.uni-duesseldorf.de>, <Julian.Schliehe-Diecks@med.uni-duesseldorf.de>, <liuyangthink@163.com>, <PaulSebastian.Jaeger@med.uni-duesseldorf.de>, <Stefanie.Geyh@med.uni-duesseldorf.de>, <Nicole.Teusch@uni-duesseldorf.de>, <Christoph.Peter@uni-duesseldorf.de>, <Sanil.Bhatia@med.uni-duesseldorf.de>, <Bodo.Levkau@med.uni-duesseldorf.de>, <Reichert@hhu.de>, <Kai.Stuehler@uni-duesseldorf.de>, <Peter.Proksch@uni-duesseldorf.de>, <Bjoern.Stork@uni-duesseldorf.de>

Dear Sebastian,
thank you very much for all your al your work and efforts with regard to the publication of our paper!
Herewith, I confirm that I fully comply with the changes made concerning the final author list.
With kind regars Rainer Haas

Bodo Levkau

Von: <Bodo.Levkau@med.uni-duesseldorf.de>
Betreff: AW: CDDIS-22-1280RR Initial Quality Check
Datum: 9. Oktober 2022 um 10:35:39 MESZ
An: <sebastian.wesselborg@uni-duesseldorf.de>

Dear Sebastian,
Herewith, I confirm that I fully comply with the changes made concerning the final author list.
Thank you
Bodo Levkau

Andreas Reichert

Von: "Reichert, Andreas" <reichert@hhu.de>
Betreff: AW: CDDIS-22-1280RR Initial Quality Check
Datum: 6. Oktober 2022 um 11:39:27 MESZ
An: Sebastian Wesselborg <sebastian.wesselborg@uni-duesseldorf.de>

Dear Sebastian,

I fully agree with these additions and the submission.

Best

Andreas

--
Prof. Dr. Andreas Reichert
Director
Institute of Biochemistry and Molecular Biology I
University Hospital Düsseldorf
Heinrich-Heine-University Düsseldorf
Postal address:
Postfach 101007
40001 Düsseldorf

Local address:
Universitätsstr. 1
Gebäude 22.03/Ebene 04
40225 Düsseldorf

Tel.: +4921181-12707 (Secretary)
Tel.: +4921181-12717
Fax.: +492118113029
Email: reichert@hhu.de
Twitter: @ReichertLab

Kai Stühler

Von: Kai Stühler <kai.stuehler@hhu.de>
Betreff: Aw: CDDIS-22-1280RR Initial Quality Check
Datum: 7. Oktober 2022 um 16:13:31 MESZ
An: Sebastian Wesselborg <sebastian.wesselborg@uni-duesseldorf.de>

Dear Sebastian,

Herewith, I confirm that I fully comply with the changes made concerning the final author list.

Best regards
Kai Stühler

Peter Proksch

Betreff: Aw: URGENT !!! Wtr: CDDIS-22-1280RR Initial Quality Check
Datum: 9. Oktober 2022 um 07:35:57 MESZ
An: Sebastian Wesselborg <sebastian.wesselborg@uni-duesseldorf.de>

Dear Prof. Wesselborg,
I agree to all changes made during the revision of the manuscript incl. the addition of further authors.
With kind regards
Peter Proksch

Björn Stork

Von: Björn Stork <bjoern.stork@uni-duesseldorf.de>
Betreff: Aw: CDDIS-22-1280RR Initial Quality Check
Datum: 6. Oktober 2022 um 11:49:54 MESZ
An: Sebastian Wesselborg <sebastian.wesselborg@uni-duesseldorf.de>

Dear Sebastian,

I agree to the changes in the author list.

Best regards,

Björn Stork

Prof. Dr. rer. nat. Björn Stork
Institute of Molecular Medicine I
University Hospital Düsseldorf
Universitätsstr. 1
Building 23.12
40225 Düsseldorf

Tel.: +49 (0)211 81-11954
E-mail: bjoern.stork@uni-duesseldorf.de
